# Supplementary material for: Selective hydrosilylation of allyl chloride with trichlorosilane
Source: Commun Chem. 2021 May 11;4:63. doi: 10.1038/s42004-021-00502-5 (PMC9814849; doi:10.1038/s42004-021-00502-5)
Supplement: Supplementary file 1 — Supplementary information [file 42004_2021_502_MOESM1_ESM.pdf]

Supplementary Information

Selective hydrosilylation of allyl chloride with trichlorosilane

Koya Inomata[1], Yuki Naganawa[1], Zhi An Wang[1,2], Kei Sakamoto[1], Kazuhiro Matsumoto[1], Kazuhiko Sato[1] & Yumiko Nakajima[1,2]*

[1] Interdisciplinary Research Center for Catalytic Chemistry, National Institute of Advanced Industrial Science and Technology (AIST), 1-1-1 Higashi, Tsukuba, Ibaraki 305-8565, Japan

[2] Graduate School of Pure and Applied Sciences, University of Tsukuba. 1-1-1 Tennoudai, Tsukuba, Ibaraki 305-8577, Japan

Email: yumiko-nakajima@aist.go.jp

Contents

1. General information S3

2. Supplementary tables S4

3. Experimental details and compound characterization data S7

4. ^{1}H, ^{13}C{^{1}H}, and ^{31}P{^{1}H} NMR spectra of synthesised compounds S17

5. Supplementary references S48

Supplementary Methods

General information

All manipulations were carried out under a nitrogen or an argon atmosphere in a glovebox or using Schlenk techniques. THF and benzene were purified by a solvent purification system (MBraun SPS-800 or a Glass Contour Ultimate Solvent System). *n*-Pentane and *n*-hexane were purchased from Kanto as "Super Dehydrated" and used as recieved. C_6D_6, CD_2Cl_2, and THF-d_8 were dried over CaH_2 and distilled prior to use. Other reagents were purchased from commercial suppliers and used without further purification unless otherwise noted. 1,2-Bis(diphenylphosphino)-3,4,5,6-tetrafluorobenzene (dppbz^F)[1], 1,2-bis(diphenylphosphino)-4,5-dimethoxybenzene (dppbz^{OMe})[2], 1,2-bis(bis(3,5-bis(trifluoromethyl)phenyl)phosphino)benzene (CF_3-dppbz)[3], 1,2-bis(bis(4-methoxyphenyl)phosphino)benzene (MeO-dppbz)[4], $[(Et_3Si)_2IrH_2(Cl)]_2$[5], $[(Et_3Si)IrH_2(SiEt_2)]_2$[5], $[Rh(\mu\text{-}Cl)(coe)_2]_2$ (coe = cyclooctene)[6], $[Rh(\mu\text{-}Cl)(cod)]_2$ (cod = 1,5-cyclooctadiene)[6], $[Rh(\mu\text{-}Cl)(dppp)]_2$ (**3**) (dppp = 1,3-bis(diphenylphosphino)propane)[7], and [Rh(Cl)(cod)(dppp)] (**4**)[8] were synthesised by following the literature procedures. ^1H, $^{13}C\{^1H\}$, $^{31}P\{^1H\}$ and $^{29}Si\{^1H\}$ NMR spectra were recorded on a Bruker AVANCE III HD 600 spectrometer. Chemical shifts are reported in δ (ppm) and are referenced to 1,4-bis-(trimethylsilyl)benzene (–4.20 ppm) for ^{29}Si and 85% H_3PO_4 (0.0 ppm) for ^{31}P, and to the residual solvent signals for ^1H and ^{13}C. High-resolution ESI mass spectra were measured on a Bruker microTOF II. Elemental analyses were performed on a Thermo Scientific FLASH2000 CHNS analyzer.

Supplementary Table 1 Reaction conditions for experiments listed in Table 1.

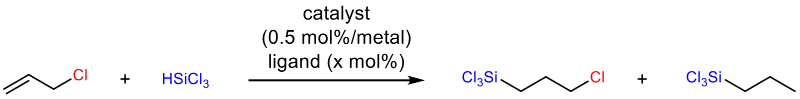

1.0 mmol   1.0 mmol   **1**   **2**

entry	catalyst (mg, μmol)		ligand (mol%, mg, μmol)	% yield (**1**/**2**)
1	Speier's catalyst[e]	(4.1, 5)	none	20/32[a]
2	Karstedt's catalyst[f]	(2.4, 2.5)[b]	none	15/13[a]
3	Karstedt's catalyst[f]	(2.4, 2.5)[b]	IMes[g] (1, 1.5, 5)[c]	53/14
4	$[Ir(\mu\text{-}Cl)(cod)]_2$[d,h]	(1.7, 2.5)	none	<5/<5
5	$[(Et_3Si)_2IrH_2(Cl)]_2$[d,i]	(2.3, 2.5)	none	<5/<5
6	$[(Et_3Si)IrH_2(SiEt_2)]_2$[d,j]	(2.7, 2.5)	none	<5/<5
7	$[Rh(\mu\text{-}Cl)(coe)_2]_2$[k]	(1.8, 2.5)	none	<5/<5
8	$[Rh(\mu\text{-}Cl)(cod)]_2$[l]	(1.2, 2.5)	none	8/<5
9	Wilkinson's catalyst[m]	(4.6, 5)	none	26/<5
10	$[Rh(\mu\text{-}Cl)(cod)]_2$[l]	(1.2, 2.5)	PPh_3 (1, 2.6, 10)	31/<5
11	$[Rh(\mu\text{-}Cl)(cod)]_2$[l]	(1.2, 2.5)	PCy_3 (1, 2.8, 10)	45/<5
12	$[Rh(\mu\text{-}Cl)(cod)]_2$[l]	(1.2, 2.5)	dppe[n] (0.5, 2.0, 5)	76/<5
13	$[Rh(\mu\text{-}Cl)(cod)]_2$[l]	(1.2, 2.5)	dppp[o] (0.5, 2.1, 5)	88/6
14	$[Rh(\mu\text{-}Cl)(cod)]_2$[l]	(1.2, 2.5)	dppbz[p] (0.5, 2.2, 5)	93/7

[a] Propene formation was detected based on ^{1}H NMR analysis of the reaction solution; 6% for entry 1 and 3% for entry 2.
[b] Xylene stock solution (45 mM, 57 μL) was used and evaporated before adding substrates.
[c] Toluene stock solution (45 mM, 114 μL) was used and evaporated before adding substrates.
[d] The utility of these iridium catalysts in the hydrosilylation of allyl chloride with $HSi(OR)_3$ or $HSiClMe_2$ was reported[9-13].

e H_2PtCl_6

f
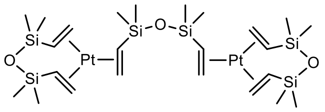

g
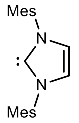

h
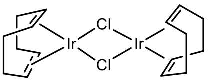

i
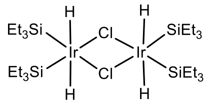

j
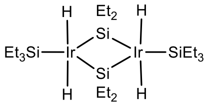

k
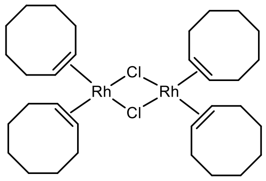

l
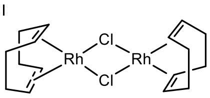

m
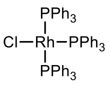

n
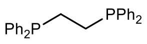

o
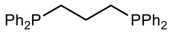

p
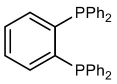

Supplementary Table 2 Reaction conditions for experiments listed in Scheme 2.

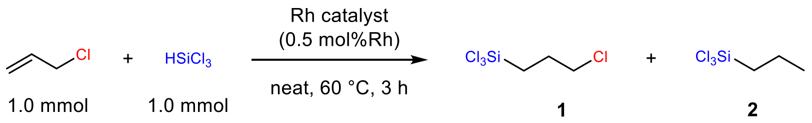

entry	catalyst (mg, μmol)		% yield (**1**/**2**)
1	**3**	(2.8, 2.5)	60/<5
2	**4**	(3.3, 5)	41/<5
3	**5**	(2.9, 2.5)	>95/<5
4	**6**	(2.1, 2.5)	<5/<5
5	**7**	(5.2, 5)	<5/<5

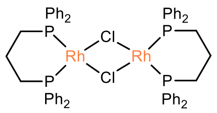

3

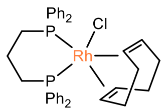

4

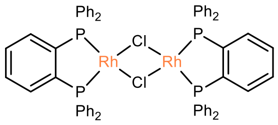

5

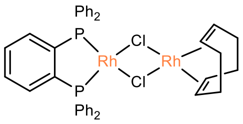

6

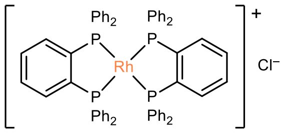

7

Supplementary Table 3 Reaction conditions for experiments listed in Table 2.

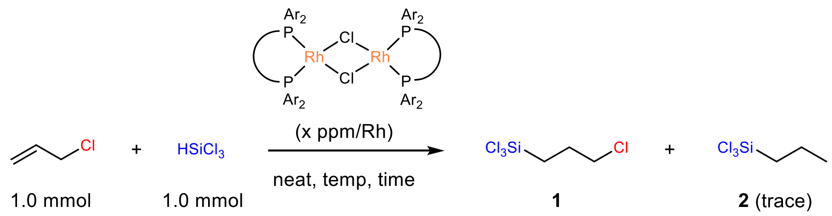

entry	Rh catalyst	x ppm/Rh	used amount (μmol)[a]	temp. (˚C)	time (h)	% yield (**1**)
1	[Rh(μ-Cl) (dppe)]$_2$	500	0.25	60	20	3
2	**3**	500	0.25	60	20	13
3	[Rh(μ-Cl) (dppb)]$_2$	500	0.25	60	20	22
4	**5**	50	0.025	60	20	>95
5	**5**	5	0.0025	60	20	11
6	**8**	50	0.025	60	20	<5
7	**9**	50	0.005	60	20	<5
8	**10**	50	0.005	60	20	<5
9	**11**	50	0.025	60	20	>95
10	**11**	50	0.025	60	1	9
11	**11**	50	0.025	60	10	73
12	**11**	50	0.025	25	20	29
13	**11**	50	0.025	40	20	39
14	**11**	5	0.0025	60	20	29
15[b]	**11**	5	0.0025	60	20	70

[a] Toluene stock solution (5 mM, 1.0 mL for entries 1-3, 100 μL for entries 4, 6-9, and 10 μL for entries 5, 10, 11) was used and toluene was removed in vacuo before adding substrates. [b] Using 3 equiv of $HSiCl_3$

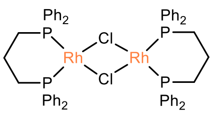

3

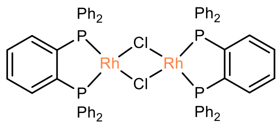

5

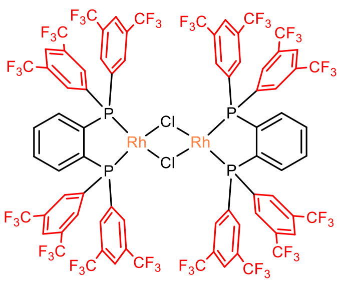

8

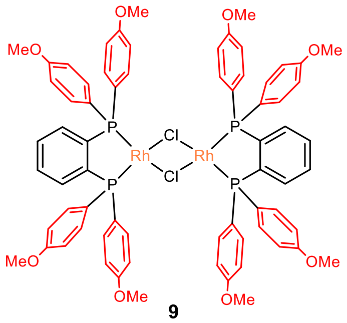

9

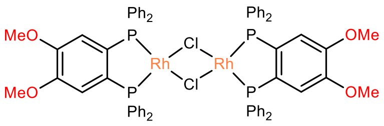

10

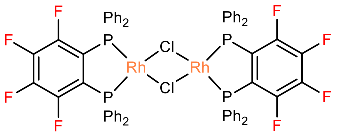

11

Experimental details and compound characterization data

General procedures for catalytic hydrosilylation of allyl chloride (Table 1, Table 2, and Fig 2c)

To a 10 mL screw vial equipped with a stir bar, were added catalyst, allyl chloride (81 μL, 1.0 mmol), and trichlorosilane (100 μL, 1.0 mmol). The mixture was stirred at 60 °C. After the reaction, formation of trichloro(3-chloropropyl)silane (**1**), trichloropropylsilane (**2**), and propene were confirmed by ^{1}H NMR spectroscopy. The yields of **1** and **2** were determined by the integral intensity ratio of the CH_2 signal at 2.78 ppm towards the signal at 2.15 ppm of mesitylene (14 μL, 0.10 mmol) as an internal standard (Fig. S1). Detailed reaction conditions for each experiment were listed in Supplementary Tables 1-3.

1: ^{1}H NMR (C_6D_6, ppm): 0.84 (m, 2H, $SiCH_2$), 1.41 (m, 2H, $SiCH_2CH_2$), 2.78 (t, 2H, $^{3}J_{HH}$ = 6.6 Hz, CH_2Cl).

2: ^{1}H NMR (C_6D_6, ppm): 0.65 (m, 3H, $SiCH_2CH_2CH_3$), 0.81 (m, 2H, $SiCH_2$), 1.24 (m, 2H, $SiCH_2CH_2$).

Propene: ^{1}H NMR (C_6D_6, ppm): 1.54 (dt, 3H, $^{3}J_{HH}$ = 6.6 Hz, $^{4}J_{HH}$ = 1.6 Hz, CH_2CHCH_3), 4.94 (m, 1H, CH_2CHCH_3), 5.00 (m, 1H, CH_2CHCH_3), 5.71 (m, 1H, CH_2CHCH_3).

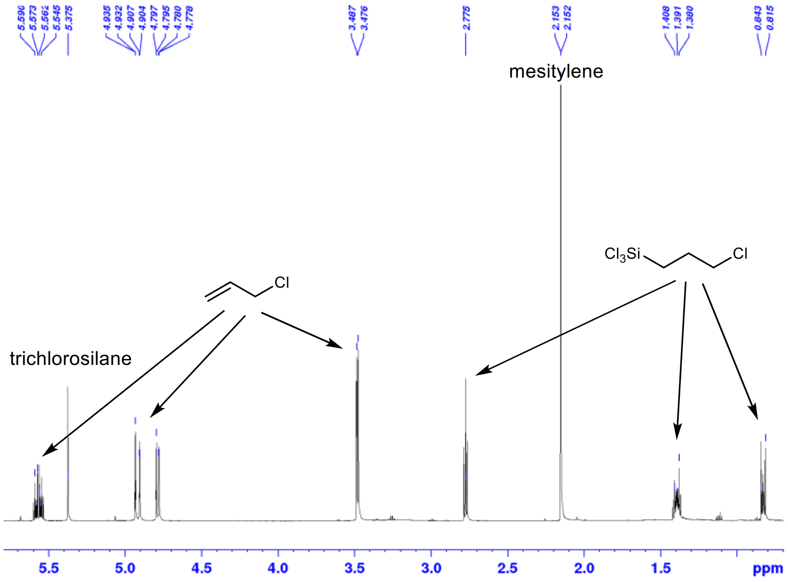

Supplementary Fig. 1 An example of ^{1}H NMR chart after reaction.

Reaction of [Rh(μ-Cl)(cod)]$_2$ with dppp (Fig. 2a)

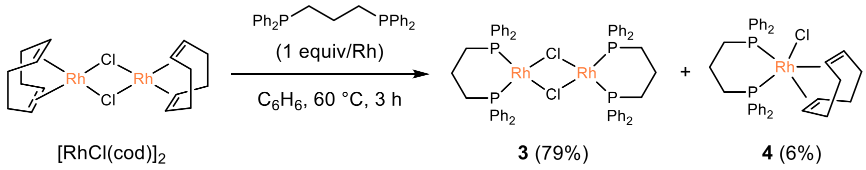

To a 6 mL screw vial containing a benzene (3 mL) solution of [Rh(μ-Cl)(cod)]$_2$ (10 mg, 0.020 mmol), was added dppp (17 mg, 0.040 mmol) at room temperature. The solution was left at 60 °C for 3 h, resulting in the oragnge solid. After all the volatiles were evaporated under vacuum, the resulting solid was completely dissolved in C_6D_6, and mesitylene (9 μL, 0.06 mmol) was added to the solution. The resulting solution was analyzed by ^{1}H NMR specrtoscopy to determine the yields of [Rh(μ-Cl)(dppp)]$_2$ (**3**) (0.016 mmol, 79%) and [RhCl(cod)(dppp)] (**4**) (0.0024 mmol, 6%). Identification of **3**[7] and **4**[7] was performed by comparing the authentic samples that were synthesised by the literature procedures.

Reaction of [Rh(μ-Cl)(cod)]$_2$ with dppbz (Fig. 2b)

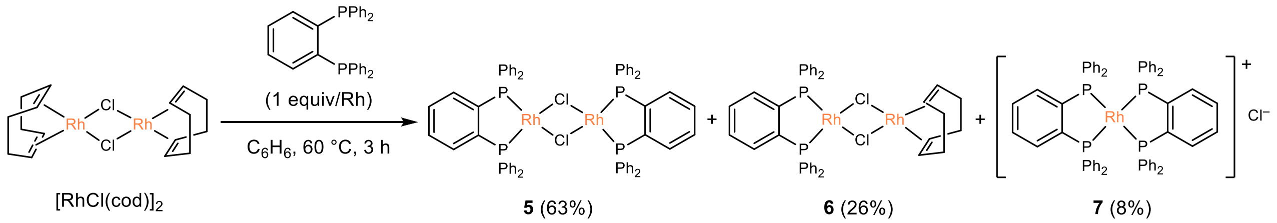

To a 6 mL screw vial containing a benzene (3 mL) solution of [Rh(μ-Cl)(cod)]$_2$ (10 mg, 0.020 mmol), was added dppbz (18 mg, 0.040 mmol) at room temperature. The solution was left at 60 °C for 3 h, resulting in the oragnge solid. After the volatiles were evaporated under vacuum, the resulting residue was dissolved in CD_2Cl_2, and mesitylene (9 μL, 0.06 mmol) was added to the solution as an internal standard. The resulting solution was analyzed by ^{1}H NMR to determine the yields of [Rh(μ-Cl)(dppbz)]$_2$ (**5**) (0.013 mmol, 63%), [(dppbz)Rh(μ-Cl)$_2$Rh(cod)] (**6**) (0.0052 mmol, 26%), and [Rh(dppbz)$_2$]Cl (**7**) (0.0034 mmol, 8%). Identification of **5**[7], **6**[8], and **7**[14] was performed by comparing the authentic samples that were alternatively synthesised by the literature procedures of the structurally similar complexes.

Synthesis of [Rh(μ-Cl)(dppbz)]$_2$ (5)

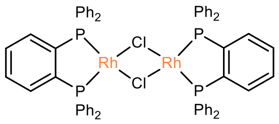

5

A THF solution (3 mL) of [Rh(μ-Cl)(cod)]$_2$ (20 mg, 0.041 mmol) was placed in a Schlenk tube (20 mL). To the solution, was added a THF (3 mL) solution containing dppbz (45 mg, 0.94 mmol) slowly over 5 min at –78 °C, and the mixture was stirred at the same temperature for 30 min. The solution was warmed to room temperature and concentrated to dryness *in vacuo*. The resulting residue was extracted with benzene (10 mL × 3) and hexane (10 mL × 3) and concentrated to dryness under vacuum to give **5** as an orange solid (41 mg, 0.035 mmol, 86%).

^{1}H NMR (C_6D_6, ppm): 6.80 (br, 4H, 3,6-CH), 6.94 (t, 16H, $^3J_{HH}$ = 7.5 Hz, Ph), 7.00 (t, 8H, $^3J_{HH}$ = 7.5 Hz, Ph), 7.40 (br, 4H, 4,5-CH), 7.94 (br, 16H, Ph). ^{13}C{^{1}H} NMR (C_6D_6, ppm): 127.3 (Ar), 128.9 (Ar), 129.7 (Ar), 131.7 (m, Ar), 133.7 (Ar), 136.4 (m, Ar), 146.3 (m, Ar). ^{31}P{^{1}H} NMR (C_6D_6, ppm): 73.6 (d, $^1J_{RhP}$ = 197 Hz). Anal. Calcd for $C_{60}H_{48}Cl_2P_4Rh_2$: C, 61.61; H, 4.14. Found: C, 61.84; H, 4.42.

Synthesis of [(dppbz)Rh(μ-Cl)$_2$Rh(cod)] (6)

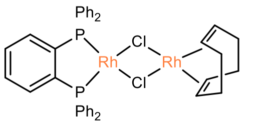

6

A THF solution (6 mL) of [Rh(μ-Cl)(cod)]$_2$ (50 mg, 0.10 mmol) was placed in a Schlenk tube (20 mL). To the solution, was added a THF (6 mL) solution containing dppbz (45 mg, 0.20 mmol) slowly over 5 min at –30 °C. After stirring at room temperature for 30 min, the solution was concentrated to the dryness *in vacuo*, and the resulting residue was washed with pentane (10 mL × 3) and benzene (10 mL × 3) and dried under vacuum. Complex **6** was obtained as an orange solid (80%, 42 mg, 0.033 mmol).

^{1}H NMR (C_6D_6, ppm): 1.40 (m, 4H, cod), 2.09 (m, 4H, cod), 4.24 (br, 4H, cod), 6.86 (m, 2H, Ar), 7.06 (m, 12H, Ar), 7.45 (m, 2H, Ar), 8.01 (m, 8H, Ar). ^{13}C{^{1}H} NMR (C_6D_6, ppm): 31.5 (cod), 76.7 (d, J = 21.9 Hz, cod), 128.6 (Ar), 129.8 (Ar), 130.6 (Ar), 132.0 (m, Ar), 134.0 (t, J = 5.8 Hz, Ar), 136.6 (Ar). One Ar signal was obscured in a residual benzene signal. ^{31}P{^{1}H} NMR (C_6D_6, ppm): 75.7 (d, $^1J_{RhP}$ = 198 Hz). Anal. Calcd for $C_{38}H_{35}Cl_2P_2Rh_2$: C, 54.90; H, 4.36. Found: C, 54.84; H, 4.31.

Synthesis of [Rh(dppbz)$_2$]Cl (7)

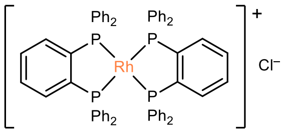

7

A benzene solution (3 mL) of [Rh(μ-Cl)(cod)]$_2$ (10 mg, 0.020 mmol) was placed in a Schlenk tube (20 mL). To the solution, was added a benzene (3 mL) solution containing dppbz (36 mg, 0.080 mmol). After stirring at room temperature for 30 min, the solution was concentrated to dryness *in vacuo* at room temperature, and the resulting residue was washed with benzene (10 mL × 3). After drying, the resulting residue was dissolved in CH_2Cl_2/hexane (2 mL/0.5 mL) and stored at –30 °C to give **7** as an orange solid (79%, 33 mg, 0.032 mmol).

^{1}H NMR (CD_2Cl_2, ppm): 6.88 (d, 16H, $^3J_{HH}$ = 6.6 Hz, Ar), 7.11 (t, 16H, $^3J_{HH}$ = 7.5 Hz, Ar), 7.38-7.46 (m, 16H, Ar). ^{13}C{^{1}H} NMR (CD_2Cl_2, ppm): 133.5 (Ar), 135.6 (Ar), 136.7 (d, J = 12 Hz, Ar), 136.8 (d, J = 19 Hz, Ar), 136.9 (m, Ar), 137.9 (Ar), 148.4 (m, Ar). ^{31}P{^{1}H} NMR (CD_2Cl_2, ppm): 62.3 (d, $^1J_{RhP}$ = 134 Hz). Anal. Calcd for $C_{60}H_{48}ClP_4Rh$: C, 69.88; H, 4.69. Found: C, 70.09; H, 4.60.

Synthesis of [Rh(μ-Cl)(CF$_3$-dppbz)]$_2$ (8)

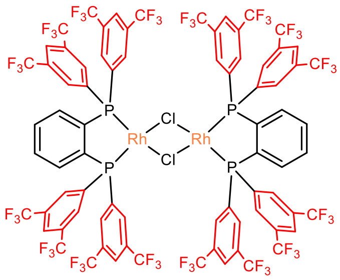

8

A benzene solution (3 mL) of [Rh(μ-Cl)(coe)$_2$]$_2$ (20 mg, 0.028 mmol) was placed in a Schlenk tube (20 mL). To the solution, was added a benzene (3 mL) solution containing CF_3-dppbz (64 mg, 0.064 mmol) slowly over 5 min at room temperature, and the mixture was stirred for 30 min. An orange precipitate was formed and filtrated to give **8** (72%, 46 mg, 0.020 mmol).

^{1}H NMR (THF-d_8, ppm): 7.70 (brm, 4H, 3,6-CH), 7.91 (brm, 4H, 4,5-CH), 8.15 (s, 8H, Ph), 8.18 (s, 16H, Ph). ^{13}C{^{1}H} NMR (THF-d_8, ppm): 123.8 (q, $^1J_{FC}$ = 273 Hz, CF_3), 125.5 (Ar), 132.7 (q, $^2J_{FC}$ = 34 Hz, Ar), 133.2 (m, Ar), 133.6 (Ar), 134.1 (Ar), 137.5 (m, Ar), 143.4 (m, Ar). ^{31}P{^{1}H} NMR (THF-d_8, ppm): 75.1 (d, $^1J_{RhP}$ = 194 Hz). Anal. Calcd for $C_{76}H_{32}Cl_2F_{48}P_4Rh_2$: C, 40.43; H, 1.43. Found: C, 40.68; H, 1.30.

Synthesis of [Rh(μ-Cl)(MeO-dppbz)]$_2$ (9)

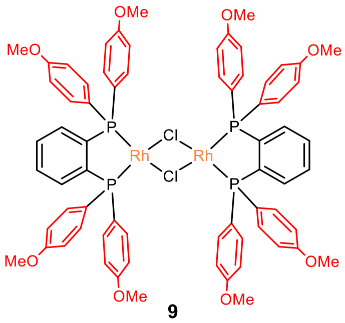

9

A THF solution (6 mL) of [Rh(μ-Cl)(cod)]$_2$ (40 mg, 0.081 mmol) was placed in a Schlenk tube (20 mL). To the solution, was added a THF (6 mL) solution containing MeO-dppbz (105 mg, 0.171 mmol) slowly over 5 min at room temperature, and the mixture was stirred for 30 min. The solution was concentrated to dryness under vacuum and the resulting residue was extract with benzene (10 mL × 3). After evaporation, **9** was obtained as an orange solid (67%, 77 mg, 0.054 mmol).

^{1}H NMR (THF-d_8, ppm): δ 3.73 (s, 24H, OMe), 6.69 (d, 16H, $^3J_{HH}$ = 4.2 Hz, Ph) 7.28 (br, 4H, 4,5-CH), 7.47 (br, 4H, 3,6-CH), 7.64 (br, 16H, Ph). ^{13}C{^{1}H} NMR (THF-d_8, ppm): 55.1 (OMe), 113.7 (Ar), 128.8 (m, Ar), 130.1 (Ar), 132.2 (m, Ar), 135.7 (Ar), 147.5 (m, Ar), 161.2 (Ar). ^{31}P{^{1}H} NMR (THF-d_8, ppm): 70.7 (d, $^1J_{RhP}$ = 199 Hz). Anal. Calcd for $C_{68}H_{64}Cl_2O_8P_4Rh_2$: C, 57.93; H, 4.58. Found: C, 57.90; H, 4.72.

Synthesis of [Rh(μ-Cl)(dppbz^{OMe})]$_2$ (10)

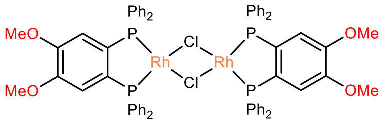

10

A THF solution (3 mL) of [Rh(μ-Cl)(cod)]$_2$ (20 mg, 0.041 mmol) was placed in a Schlenk tube (20 mL). To the solution, was added a benzene (3 mL) solution containing dppbz^{OMe} (47 mg, 0.093 mmol) slowly over 5 min at –30 °C. After stirring at room temperature for 30 min, the solution was concentrated to dryness *in vacuo* at room temperature, and the resulting residue was extract with benzene (10 mL × 3). After benzene was removed by evaporation, the obtained solid was washed with hexane (10 mL × 3) to give **10** as an orange solid (80%, 42 mg, 0.033 mmol).

^{1}H NMR (THF-d_8, ppm): 3.57 (s, 12H, OMe), 6.89 (t, 4H, J = 3.2 Hz, 3,6-CH) 7.18 (t, 16H, $^3J_{HH}$ = 7.5 Hz, Ph), 7.24 (t, 8H, $^3J_{HH}$ = 7.3 Hz, Ar), 7.72 (br, 16H, Ar). ^{13}C{^{1}H} NMR (THF-d_8, ppm): 56.0 (OMe), 113.7 (m, Ar), 128.4 (m, Ar), 129.6 (Ar), 134.4 (m, Ar), 137.8 (m, Ar), 138.8 (m, Ar), 152.5 (Ar). ^{31}P{^{1}H} NMR

(THF-d_8, ppm): 72.4 (d, $^1J_{RhP}$ = 197 Hz). Anal. Calcd for $C_{64}H_{56}Cl_2O_4P_4Rh_2$: C, 59.60; H, 4.38. Found: C, 59.70; H, 4.72.

Synthesis of [Rh(μ-Cl)(dppbz^F)]$_2$ (11)

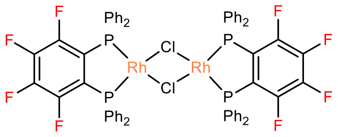

11

A benzene solution (3 mL) of [Rh(μ-Cl)(coe)$_2$]$_2$ (20 mg, 0.028 mmol) was placed in a Schlenk tube (20 mL). To the solution, was added a benezene (3 mL) solution containing dppbz^F (33 mg, 0.064 mmol) slowly over 5 min at room temperature, and the mixture was stirred for 30 min at the temperature. The solution was concentrated to the dryness *in vacuo*, and the resulting residue was extract with benzene (10 mL × 3). After evaporation, the obtained solid was washed with hexane (10 mL × 3) to give **11** as an orange solid (72%, 26 mg, 0.020 mmol).

^{1}H NMR (THF-d_8, ppm): 7.26 (t, 16H, $^3J_{HH}$ = 7.5 Hz, Ph), 7.37 (t, 8H, $^3J_{HH}$ = 7.5 Hz, Ph), 7.78 (m, 16H, Ph). ^{13}C{^{1}H} NMR (THF-d_8, ppm): 128.4 (t, J = 4.5 Hz, Ar), 129.0 (Ar), 130.4 (Ar), 134.0 (t, J = 5.6 Hz, Ar), 134.2 (d, J = 24 Hz, Ar), 143.4 (brd, $^1J_{CF}$ = 259 Hz, Ar), 147.7 (brd, $^1J_{CF}$ = 253 Hz, Ar). ^{31}P{^{1}H} NMR (THF-d_8, ppm): 80.3 (d, $^1J_{RhP}$ = 200 Hz). Anal. Calcd for $C_{60}H_{40}Cl_2F_8P_4Rh_2$: C, 54.86; H, 3.07. Found: C, 54.54; H, 3.35.

Reaction of [Rh(μ-Cl)(dppbz^F)]$_2$ (11) with allyl chloride (Fig. 3a)

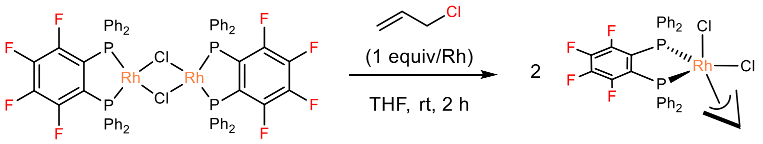

11 **12** (74%)

A THF (2 mL) solution of **11** (50 mg, 0.045 mmol) was placed in a Schlenk tube (20 mL). Allyl chloride (69 mg, 0.90 mmol) was added to the solution, and the mixture was stirred at room temperature for 2 h, resulting in the formation of yellow precipitates. After filtration, the obtained solid was washed with hexane (2 mL × 3) and THF (2 mL × 3) to give [Rh(π-allyl)Cl$_2$(dpppbz^F)] (**12**) (44 mg, 0.069 mmol, 74%).

^{1}H NMR (CD_2Cl_2, ppm): 2.91 (d, $^3J_{HH}$ = 9.0 Hz, 2H, allyl), 4.32 (dt, $^3J_{HH}$ = 7.8 Hz, $^3J_{HH}$ = 3.3 Hz, 1H, allyl), 4.58 (dt, $^3J_{HH}$ = 14 Hz, $^3J_{HH}$ = 4.2 Hz, 1H, allyl), 5.03 (m, 1H, allyl), 7.36 (m, 8H, Ph), 7.50 (m, 6H, Ph), 7.57 (dd, $^3J_{HH}$ = 8.4 Hz, $^3J_{HH}$ = 6.6 Hz, 2H, Ph), 7.80 (dd, $^3J_{HH}$ = 12 Hz, $^3J_{HH}$ = 7.8 Hz, 4H, Ph).

$^{13}C\{^1H\}$ NMR (CD_2Cl_2, ppm): 55.9 (d, J = 11 Hz, allyl), 81.7 (dt, J = 11 Hz, J = 3.5 Hz, allyl), 107.8 (d, J = 4.0 Hz, allyl). 125.3 (brm, Ar), 127.4 (d, J = 62 Hz, Ar), 128.1 (d, J = 12 Hz, Ar), 129.4 (d, J = 11 Hz, Ar), 129.9 (d, J = 49 Hz, Ar), 131.8 (d, J = 2.9 Hz, Ar), 132.2 (d, J = 2.4 Hz, Ar), 132.6 (d, J = 10 Hz, Ar), 133.9 (d, J = 9.2 Hz, Ar), 143.9 (brd, $^1J_{CF}$ = 268 Hz, Ar), 149.0 (brd, $^1J_{CF}$ = 255 Hz, Ar). $^{31}P\{^1H\}$ NMR (CD_2Cl_2, ppm): 63.0 (brd, $^1J_{RhP}$ = 76 Hz). Anal. Calcd for $C_{33}H_{25}Cl_2F_4P_2Rh$: C, 54.05; H, 3.44. Found: C, 54.19; H, 3.44.

Reaction of [Rh(π-allyl)Cl_2(dpppbz^F)] (12) with $HSiCl_3$ (Fig. 3b)

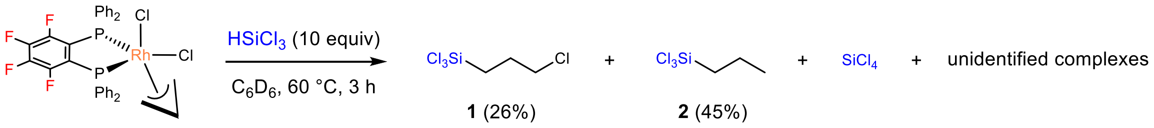

12

A J-young NMR tube was charged with a C_6D_6 (0.5 mL) solution of **12** (3.7 mg, 0.0050 mmol). To the solution, was added $HSiCl_3$ (5 μL, 0.05 mmol) at room temperature. The solution was left at 60 °C for 3 h, and then mesitylene (6 μL, 0.06 mmol) was added. Formation of **1** (26%) and **2** (45%) were confirmed by ^1H NMR spectroscopy. Formation of hydrido species, which exhibit hydride signals at –15.01 (dt, J = 21, 7.2 Hz), –15.73 (dt, J = 25, 9.0 Hz), and –16.85 (m) ppm with the integral intensity of 34 : 13 : 15, were observed in the ^1H NMR spectrum. In the $^{31}P\{^1H\}$ NMR, several signals were observed in the range of 26.1 ppm to 84.2 ppm. The resulting complexes underwent further transformation under vacuum conditions to form a complicated mixture containing [Rh(μ-Cl)(dppbz^F)]$_2$ (**11**) and several unidentification species. Therefore, identification of the resulting hydride species were not successfull. The observed hydride signals at –15.01 and –16.85 ppm also appeared on the ^1H NMR monitoring of the reaction of [Rh(μ-Cl)(dppbz^F)]$_2$ (**11**) with $HSiCl_3$ (20 equiv), which resulted in the formation of a complex mixture of unidentified complexes. The identification of these resulting complexes was not successful since these complexes were easily transferred to [Rh(μ-Cl)(dppbz^F)]$_2$ (**11**) and unidentified complexes after evaporation, probably via reductive elimination.

Reaction of [Rh(π-allyl)Cl_2(dpppbz^F)] (12) with cinnamyl chloride (Fig. 3d)

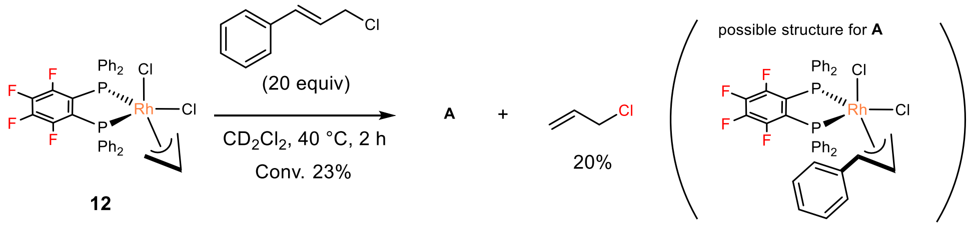

A J-young NMR tube was charged with a CD_2Cl_2 (0.5 mL) solution of **12** (4 mg, 0.006 mmol). To the solution, were added cinnamyl chloride (15 μL, 0.11 mmol) and mesitylene (1 μL, 0.01 mmol) at room temperature. The solution was left at 40 °C for 2 h, formation of free allyl chloride (0.001 mmol, 20%) was detected by ^{1}H NMR spectroscopy. The conversions of **12** and cinnamyl chloride were determined as 23% and 19%, respectively. In the ^{31}P{^{1}H} NMR spectrum, a new doublet of doublets signal and the signal assignable to unreacted **12** were observed at 66.7 ppm ($^1J_{RhP}$ = 130 Hz, $^2J_{PP}$ = 8.9 Hz) and at 63.0 ppm with the integral intensity of 66/34, respectively.

Experimental data of **A**

^{31}P{^{1}H} NMR (CD_2Cl_2, ppm): 66.7 ppm ($^1J_{RhP}$ = 130 Hz, $^2J_{PP}$ = 8.9 Hz). HRMS (ESI) Calculated: ($C_{39}H_{29}ClF_4P_2Rh$) 773.4019 ([M−Cl$^-$]$^+$), Found: 773.0426.

Synthesis of [Rh(π-allyl)Cl_2(dppp)] (13)

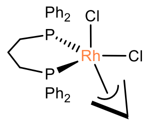

13

A THF (2 mL) solution of [Rh(μ-Cl)(dppp)]$_2$ (**3**) (50 mg, 0.045 mmol) was placed in a Schlenk tube (20 mL). Allyl chloride (69 mg, 0.90 mmol) was added to the solution, and the mixture was stirred at room temperature for 2 h, resulting in the formation of yellow precipitates. After filtration, the obtained solid was washed with hexane (2 mL × 3) and THF (2 mL × 3) to give **13** (42 mg, 0.066 mmol, 74%).

^{1}H NMR (CD_2Cl_2, −30 °C, ppm): 1.34 (vsext, 1H, J = 11 Hz, C_3H_6), 1.75 (m, 1H, C_3H_6), 2.26 (m, 1H, C_3H_6), 2.57 (td, 1H, $^2J_{HH}$ = 16 Hz, $^3J_{HH}$ = 4.4 Hz, C_3H_6), 2.78 (m, 1H, C_3H_6), 3.08 (d, 1H, $^3J_{HH}$ = 6.6 Hz, C_3H_6), 3.12 (dd, 1H, $^3J_{HH}$ = 6.9 Hz, $^3J_{HH}$ = 1.5 Hz, allyl), 3.54 (m, 1H, allyl) 3.68 (d, 1H, $^3J_{HH}$ = 9.0 Hz, allyl), 4.15 (dd, 1H, $^2J_{HH}$ = 13.5 Hz, $^3J_{HH}$ = 7.8 Hz, allyl), 4.90 (*m*, 1H, allyl), 6.84 (dd, 2H, $^3J_{HH}$ = 8.2 Hz, $^3J_{HH}$ = 1.8 Hz, Ph), 7.00 (m, 2H, Ph), 7.30 (m, 2H, Ph), 7.35 (m, 2H, Ph), 7.47 (m, 2H, Ph), 7.55 (t, 1H, $^3J_{HH}$ = 7.3 Hz, Ph), 7.62 (t, 2H, $^3J_{HH}$ = 6.8 Hz, Ph), 7.68 (t, 1H, $^3J_{HH}$ = 6.9 Hz, Ph), 7.73 (t, 2H, $^3J_{HH}$ = 8.8

Hz, Ph), 7.78 (brm, 2H, Ph), 7.84 (brm, 2H, Ph). $^{13}C\{^1H\}$ NMR (CD_2Cl_2, −30 °C, ppm): 18.6 (C_3H_6), 25.1 (dd, J = 27 and 4.4 Hz, C_3H_6), 26.3 (dd, J = 38 and 3.3 Hz, C_3H_6), 58.6 (d, J = 18 Hz, allyl), 74.6 (dd, J = 31 and 3.8 Hz, allyl), 107.3 (d, J = 3.6 Hz, allyl), 127.4 (d, J = 16 Hz, Ar), 128.1 (d, J = 16 Hz, Ar), 128.4 (d, J = 16 Hz, Ar), 128.6 (d, J = 81 Hz, Ar), 129.0 (d, J = 16 Hz, Ar), 129.6 (Ar), 130.3 (d, J = 88 Hz, Ar), 131.0 (Ar), 131.1 (Ar), 131.6 (Ar), 132.4 (br, Ar), 132.6 (d, J = 11 Hz, Ar), 133.2 (d, J = 14 Hz, Ar), 133.3 (d, J = 68 Hz, Ar), 133.7 (d, J = 17 Hz, Ar), 135.5 (d, J = 83 Hz, Ar). $^{31}P\{^1H\}$ NMR (CD_2Cl_2, −30 °C, ppm): 3.5 (dd, $^1J_{RhP}$ = 130 Hz, $^3J_{PP}$ = 29 Hz), 31.3 (dd, $^1J_{RhP}$ = 103 Hz, $^3J_{PP}$ = 29 Hz). Anal. Calcd for $C_{30}H_{31}Cl_2P_2Rh$: C, 57.44; H, 4.98. Found: C, 57.26; H, 5.14.

Reaction of [Rh(π-allyl)Cl_2(dppp)] (13) with $HSiCl_3$ (Fig. 3e)

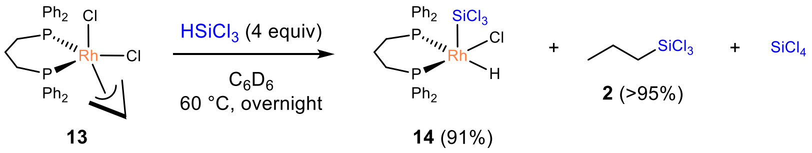

To a C_6D_6 (0.5 mL) suspension of **13** (3.1 mg, 0.0049 mmol), was added $HSiCl_3$ (20 μL, 0.20 mmol) at room temperature. The solution was left at 60 °C for overnight. After the reaction, mesitylene (2 μL, 0.02 mmol) as an internal standard was added to the solution. Based on ^1H NMR analysis, formation of [Rh(Cl)(H)($SiCl_3$)(dppp)] (**14**) (0.0044 mmol, 90%), **2** (>0.0047 mmol, >95%), and propene (trace) was confirmed. In the $^{29}Si\{^1H\}$ NMR spectrum, $SiCl_4$ was observed at –18.9 ppm. Formation of **14** was confirmed by comparing the NMR of the alternatively synthesised authentic sample (vide infra).

Alternative synthetic path for 14: Reaction of [Rh(μ-Cl)(dppp)]$_2$ with $HSiCl_3$

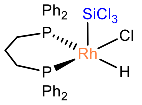

14

A toluene solution (5 mL) of [Rh(μ-Cl)(dppp)]$_2$ (**3**) (50 mg, 0.045 mmol) was placed in a Schlenk tube (20 mL). $HSiCl_3$ (27 μL , 0.27 mmol) was added to the solution, and the mixture was stirred at room temperature for 2 h. The solution was concentrated to the dryness *in vacuo*. The resulting residue was dissolved in CH_2Cl_2/hexane (2 mL/0.5 mL) and stored at –30 °C to give **14** as an orange solid (55 mg, 0.079 mmol, 88%).

^{1}H NMR (C_6D_6, ppm): –6.17 (dd, $^{2}J_{HP}$ = 115 Hz, $^{1}J_{HRh}$ = 15.6 Hz, 1H, RhH, Rather small $^{2}J_{HPcis}$ [normally 4-18 Hz][15] is obscured), 1.40 (m, 2H, C_3H_6), 2.22 (m, 2H, C_3H_6), 2.32 (m, 2H, C_3H_6), 6.95 (m, 12H, Ph), 7.40 (m, 8H, Ph). ^{13}C{^{1}H} NMR (C_6D_6, ppm): 18.9 (s, C_3H_6), 23.4 (d, $^{1}J_{CP}$ = 21 Hz, C_3H_6), 27.2 (m, C_3H_6), 128.5 (d, $^{2}J_{CP}$ = 10 Hz, Ar), 129.3 (d, $^{2}J_{CP}$ = 10 Hz, Ar), 130.8 (Ar), 131.1 (Ar), 132.3 (Ar), 132.4 (d, $^{3}J_{CP}$ = 20 Hz, Ar), 132.5 (Ar), 134.1 (d, $^{3}J_{CP}$ = 20 Hz, Ar). ^{31}P{^{1}H} NMR (C_6D_6, ppm): 8.17 (dd, $^{1}J_{RhP}$ = 102 Hz, $^{2}J_{PP}$ = 34 Hz), 9.52 (dd, $^{1}J_{RhP}$ = 97 Hz, $^{2}J_{PP}$ = 34 Hz). Anal. Calcd for $C_{27}H_{27}Cl_4P_2RhSi$: C, 47.26; H, 3.97. Found: C, 47.05; H, 3.92.

Gram scale synthesis of 1 (Fig. 5)

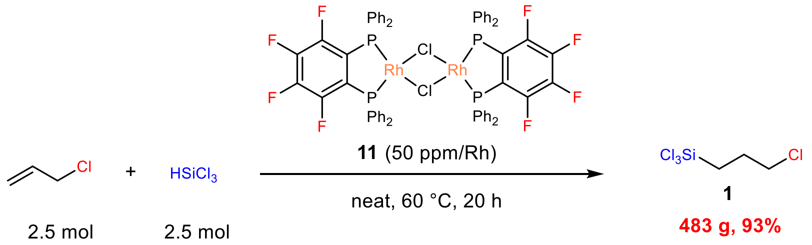

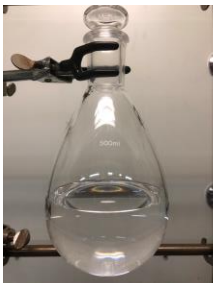

A rhodium catalyst [Rh(μ-Cl)(dppbz^{F})]$_2$ (**11**) (80 mg, 0.060 mmol) was placed in a three-necked flask (3 L) with condenser. Allyl chloride (187 g, 2.45 mol) and trichlorosilane (332 g, 2.45 mol) were added to the flask, and the mixture was stirred at 60 °C for 20 h. After distillation of the resulting solution under reduced pressure (36 hPa, 80 °C), analytically pure **1** was obtained as a colorless liquid (483 g, 2.28 mol, 93%).

^1H, $^{13}C\{^1H\}$, and $^{31}P\{^1H\}$ NMR spectra of synthesised compounds

Supplementary Fig. 2 ^1H NMR spectra of $[Rh(\mu\text{-}Cl)(dppbz)]_2$ (5) (C_6D_6).

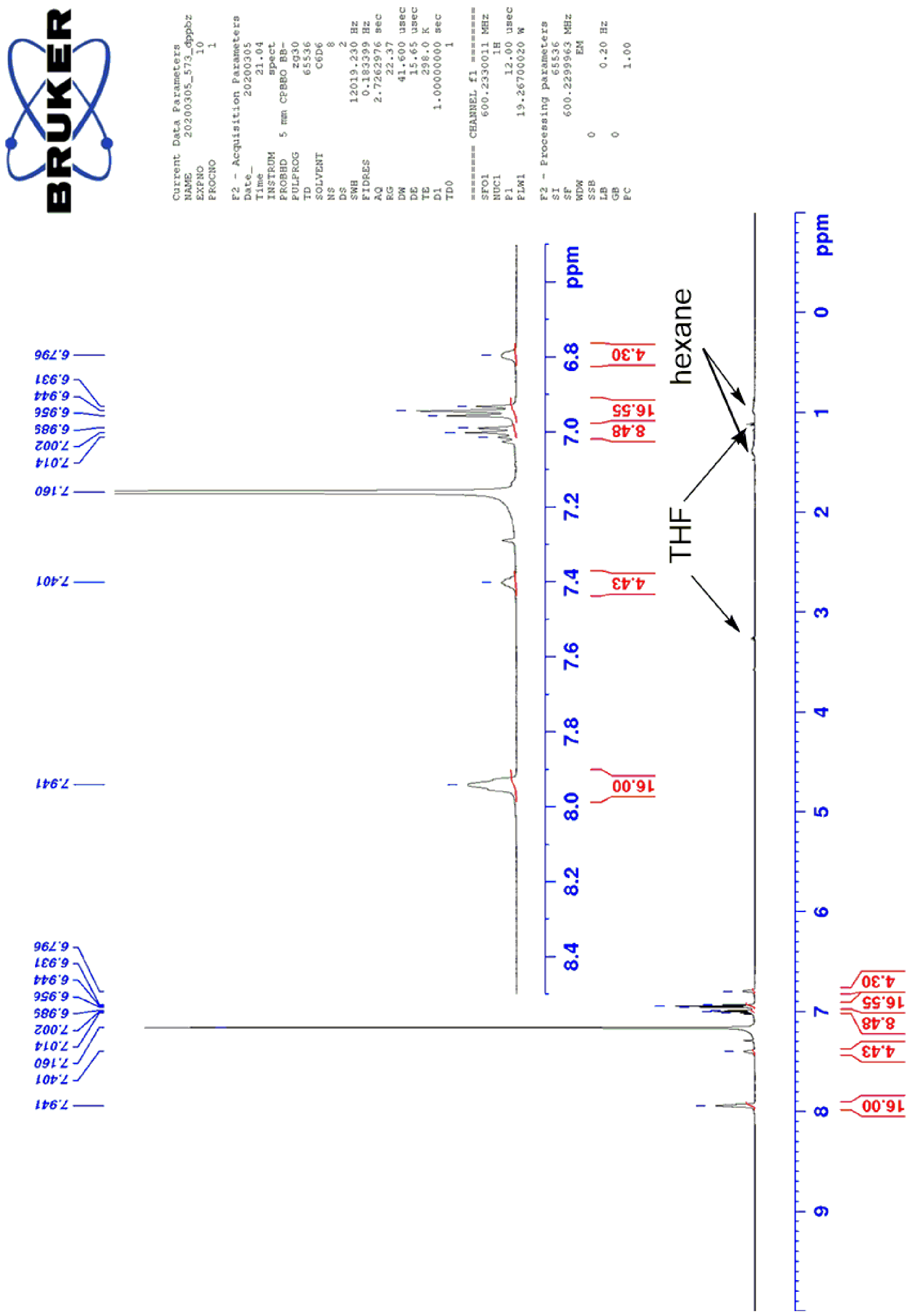

Supplementary Fig. 3 $^{13}C\{^1H\}$ NMR spectra of [Rh(μ-Cl)(dppbz)]$_2$ (5) (C_6D_6).

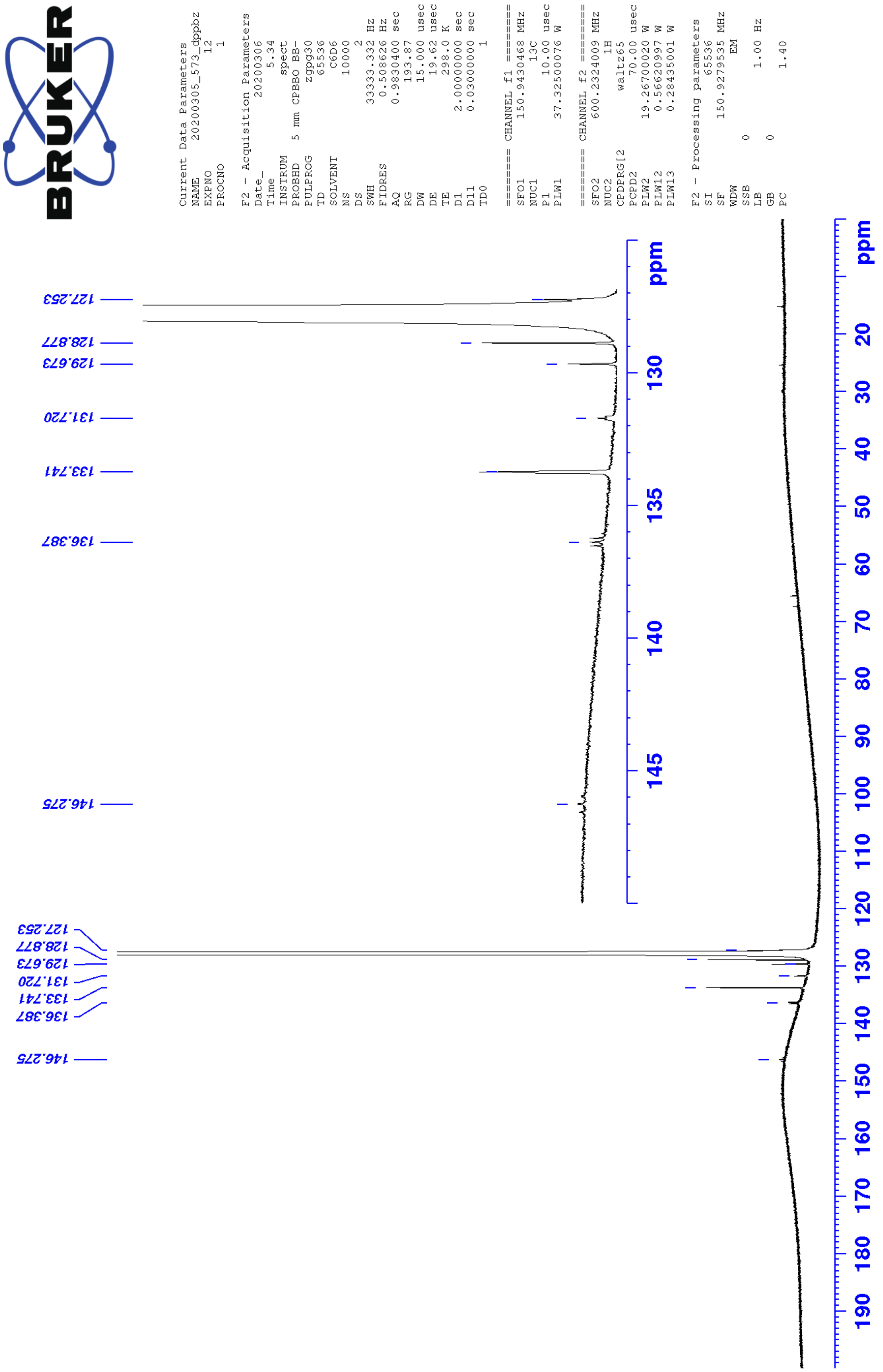

Supplementary Fig. 4 $^{31}P\{^1H\}$ NMR spectra of $[Rh(\mu\text{-}Cl)(dppbz)]_2$ (5) (C_6D_6).

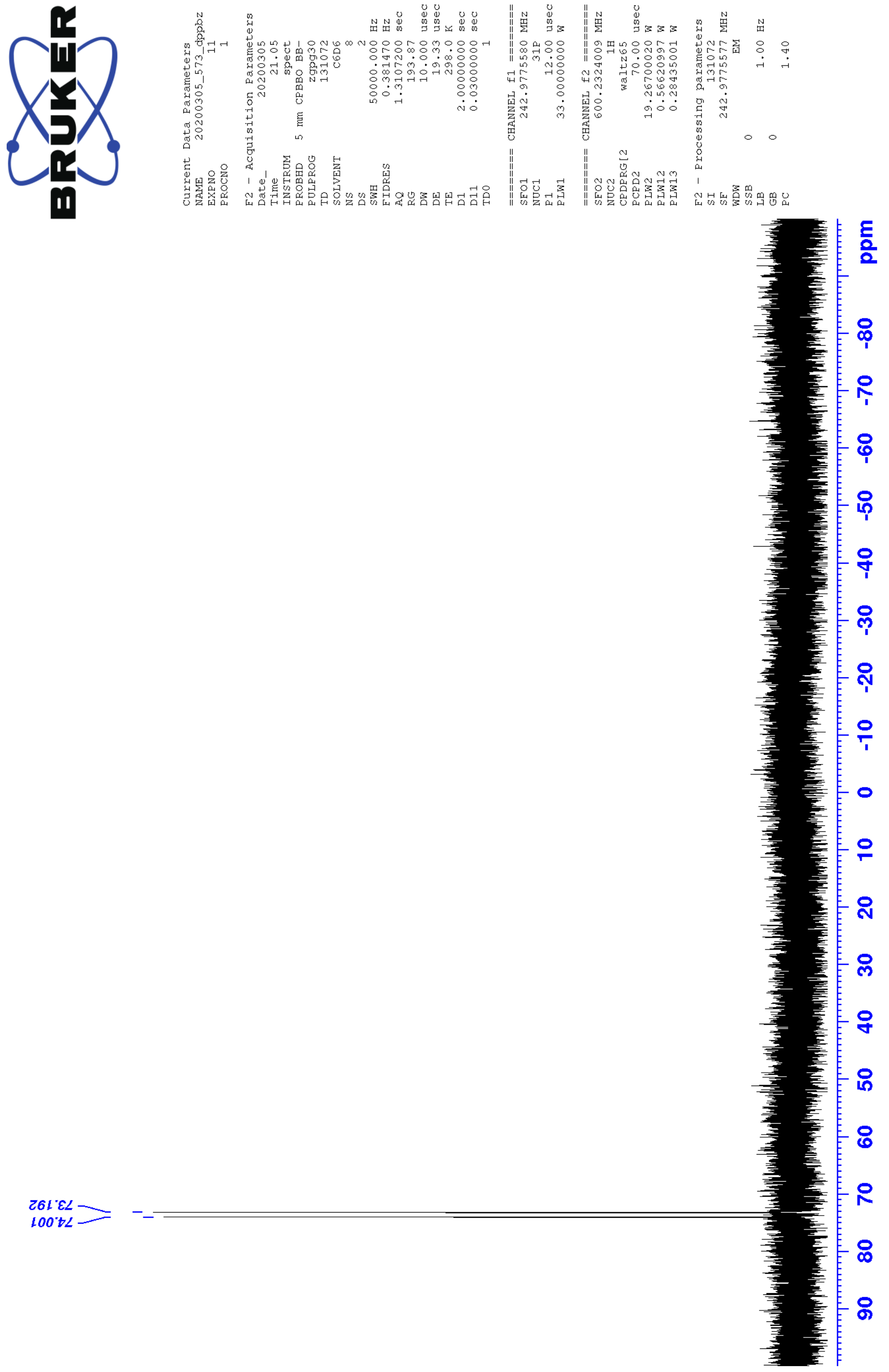

Supplementary Fig. 5 ^{1}H NMR spectra of [(dppbz)Rh(μ-Cl)$_2$Rh(cod)] (6) (C_6D_6).

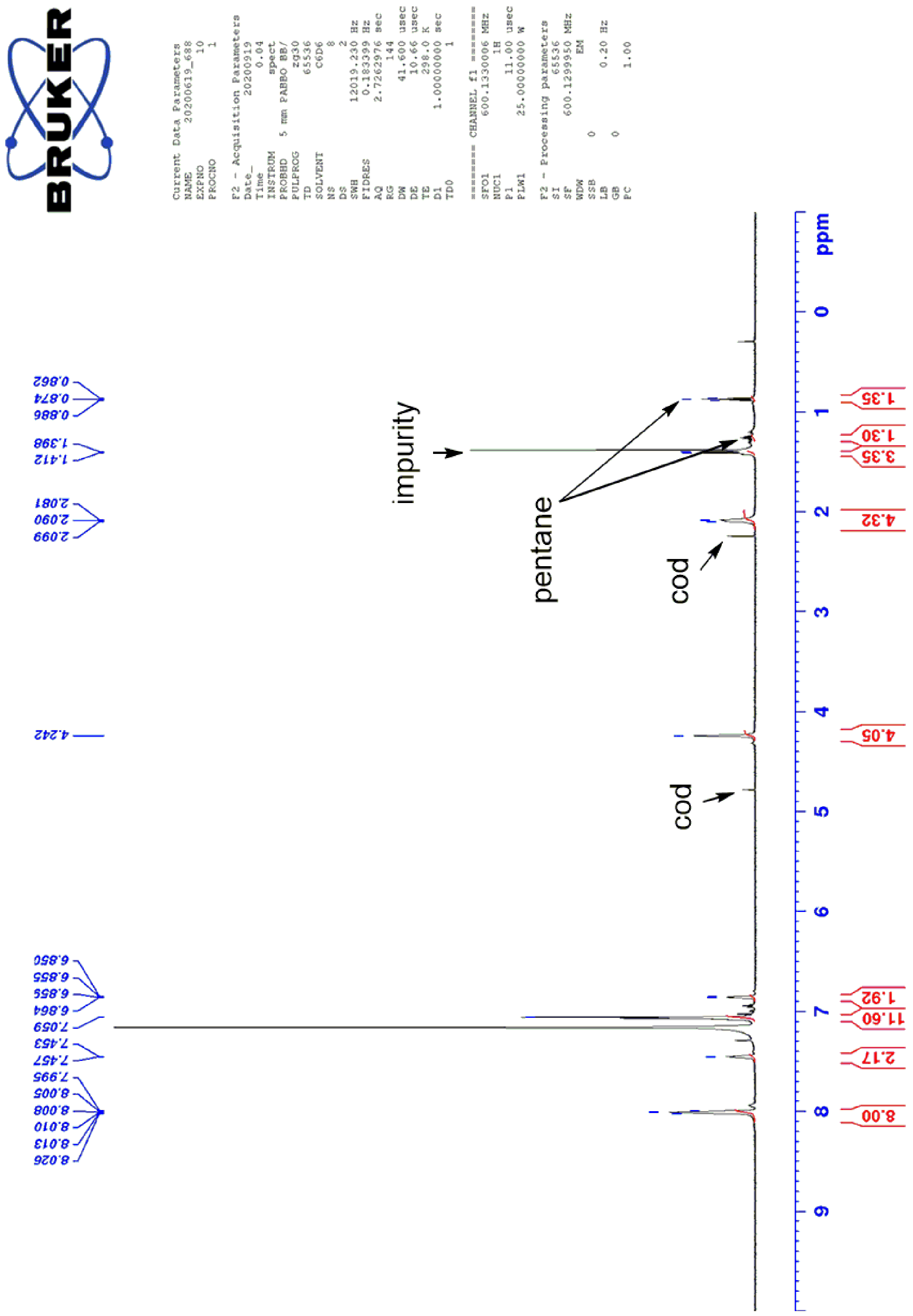

Supplementary Fig. 6 $^{13}C\{^1H\}$ NMR spectra of [(dppbz)Rh(μ-Cl)$_2$Rh(cod)] (6) (C_6D_6).

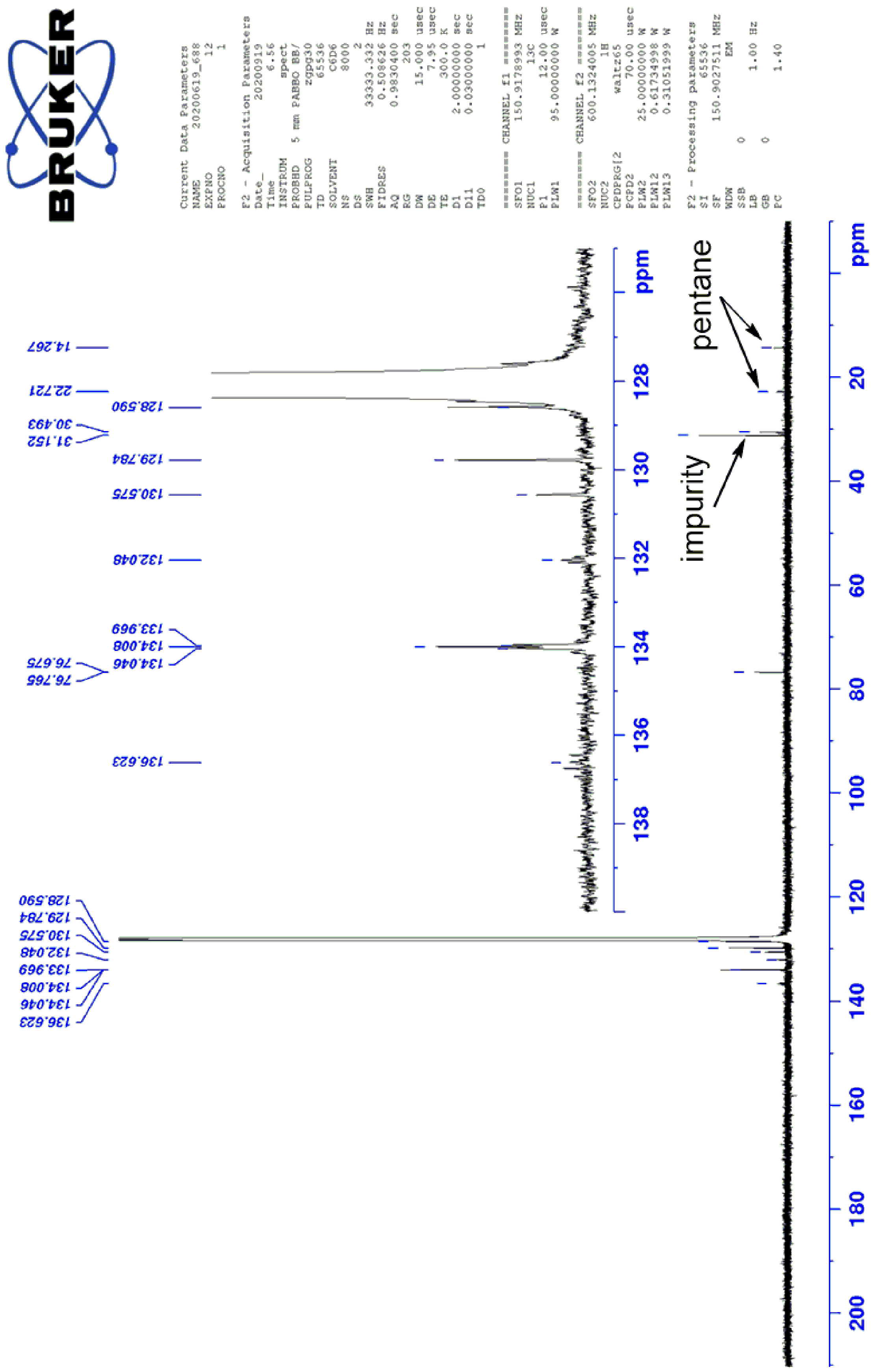

Supplementary Fig. 7 $^{31}P\{^{1}H\}$ NMR spectra of [(dppbz)Rh(μ-Cl)$_2$Rh(cod)] (6) (C_6D_6).

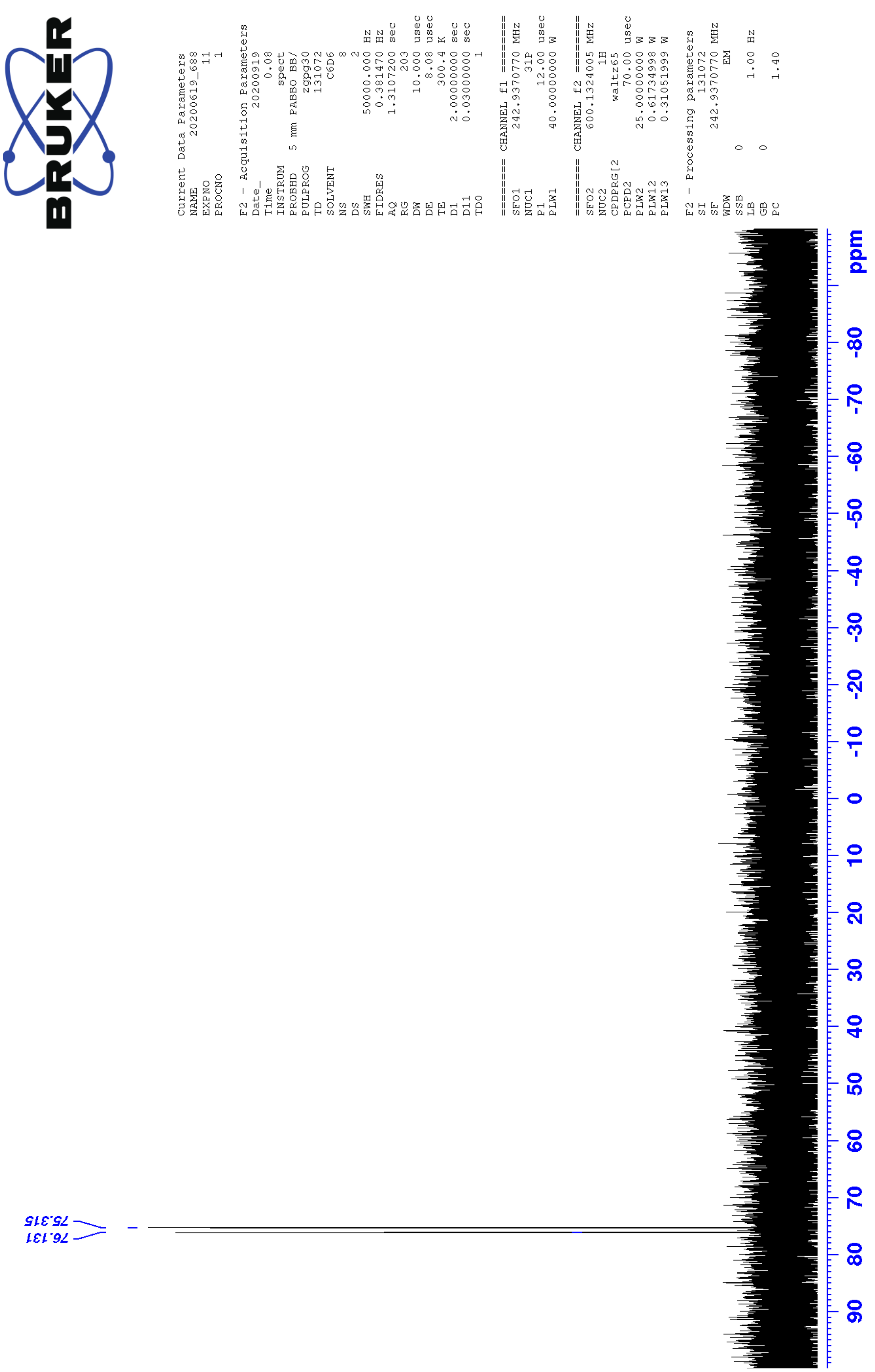

Supplementary Fig. 8 ^{1}H NMR spectra of $[Rh(dppbz)_2]Cl$ (7) (CD_2Cl_2).

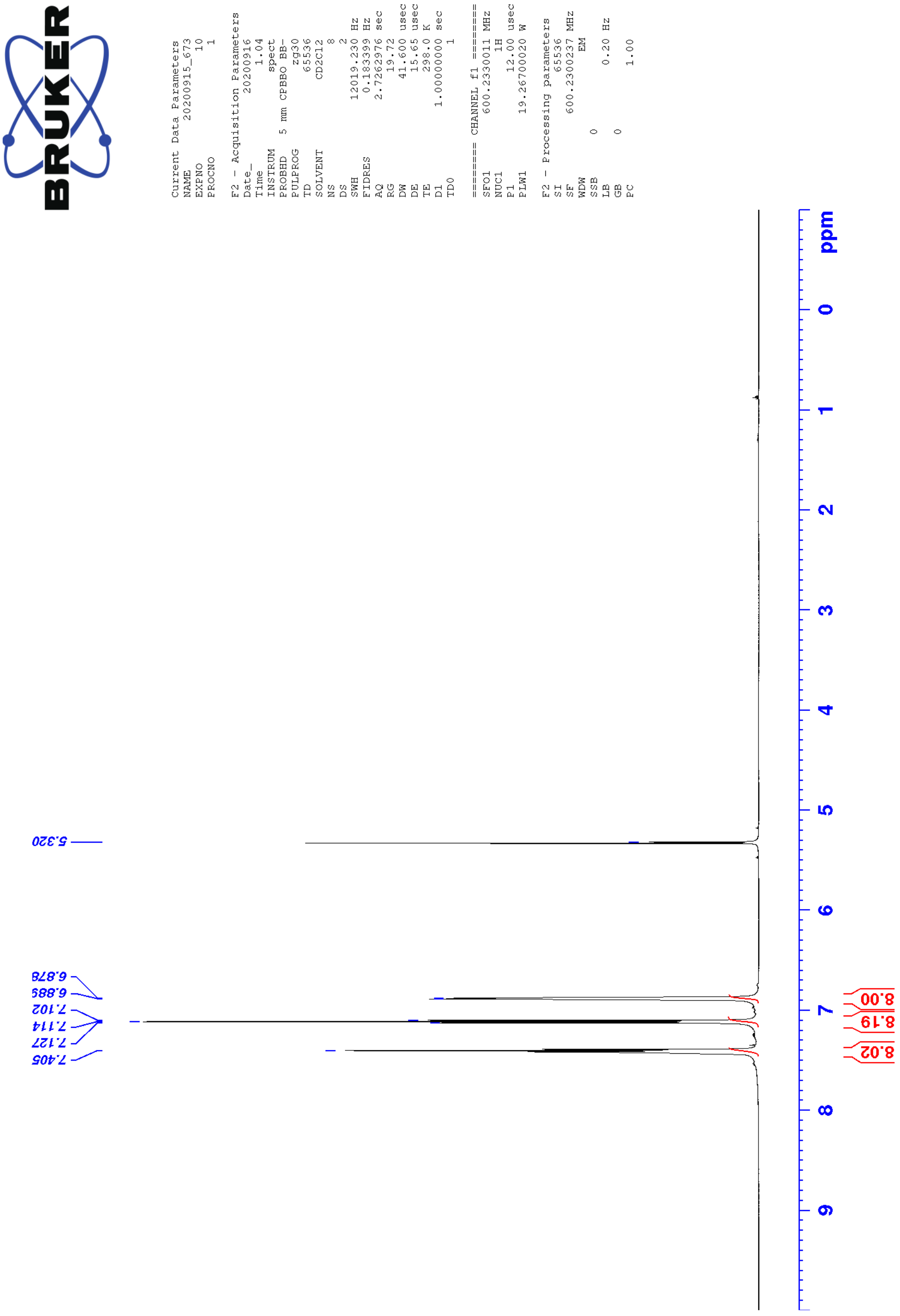

Supplementary Fig. 9 $^{13}C\{^{1}H\}$ NMR spectra of $[Rh(dppbz)_2]Cl$ (7) (CD_2Cl_2).

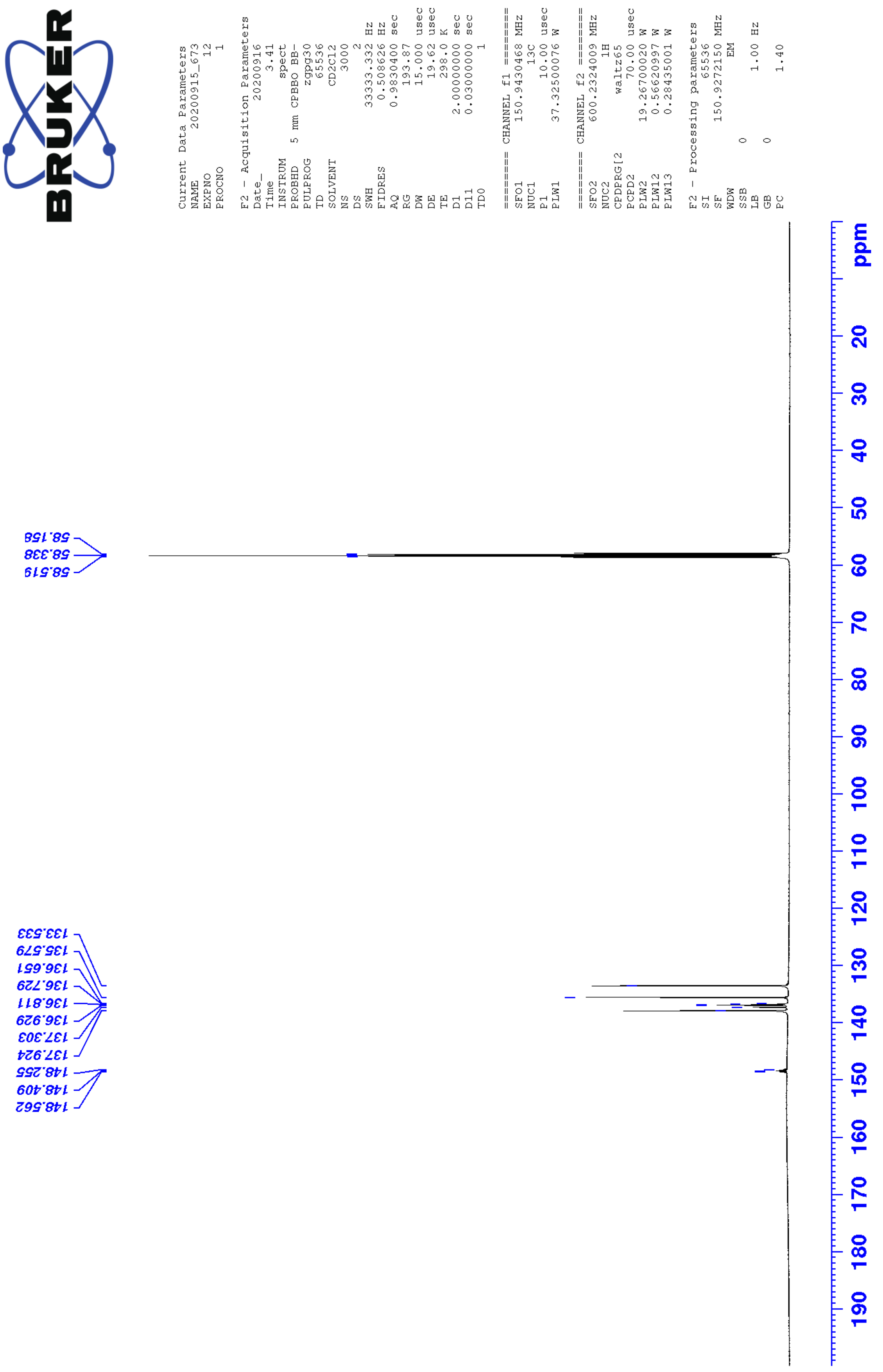

Supplementary Fig. 10 $^{31}P\{^{1}H\}$ NMR spectra of $[Rh(dppbz)_2]Cl$ (7) (CD_2Cl_2).

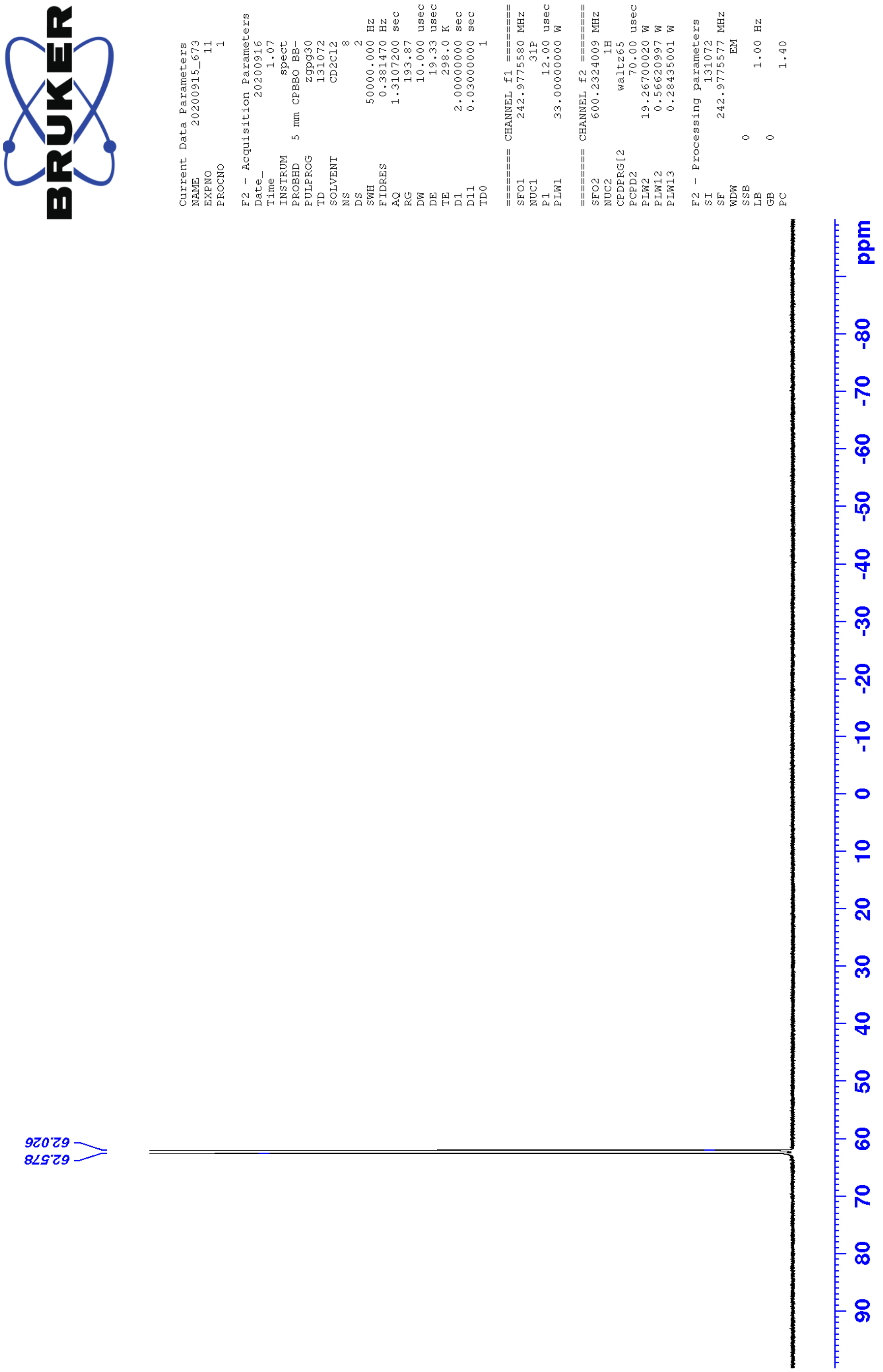

Supplementary Fig. 11 ^{1}H NMR spectra of [Rh(μ-Cl)(CF_3-dppbz)]$_2$ (8) (THF-d_8).

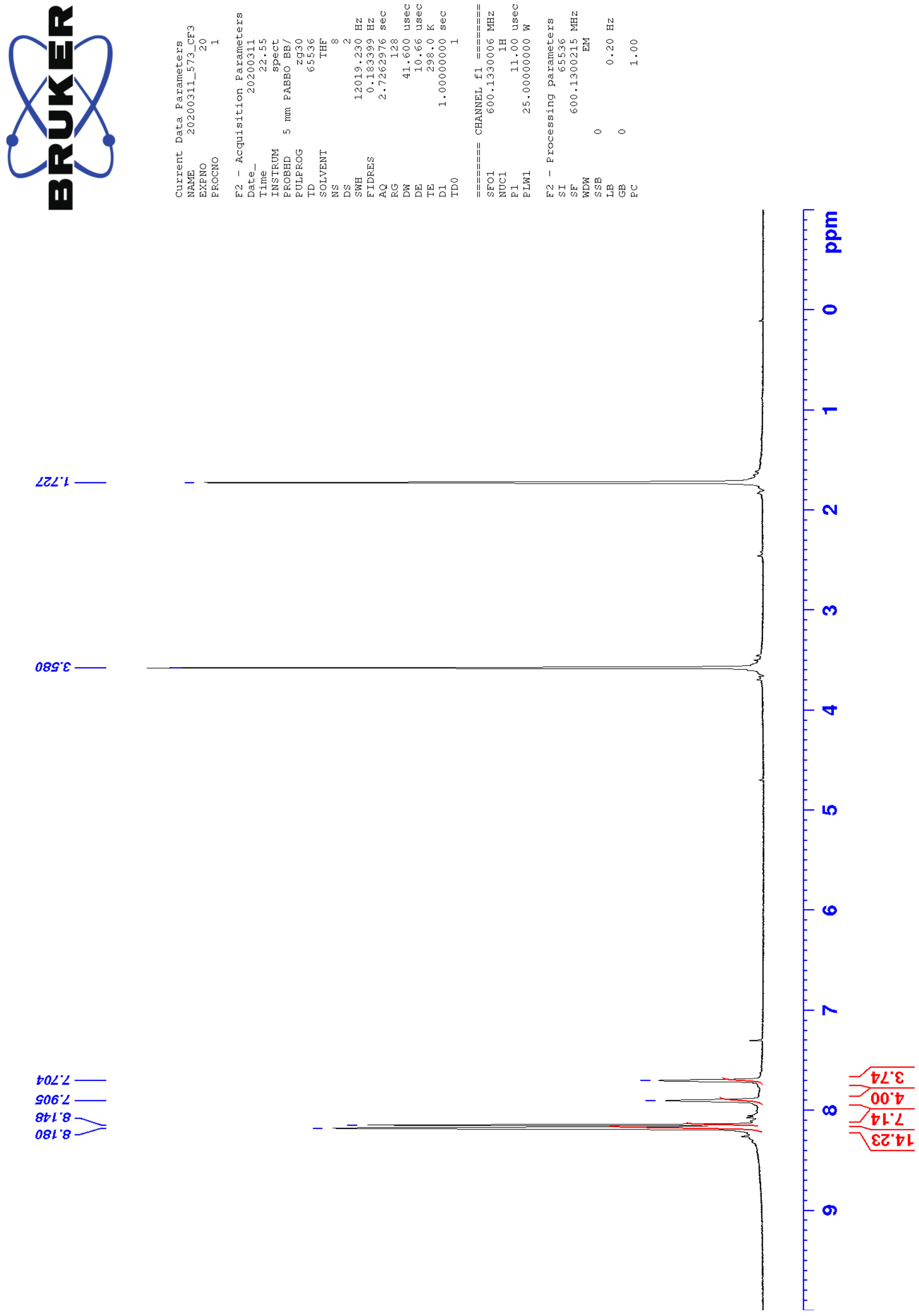

Supplementary Fig. 12 $^{13}C\{^{1}H\}$ NMR spectra of $[Rh(\mu\text{-}Cl)(CF_3\text{-}dppbz)]_2$ (8) (THF-d_8).

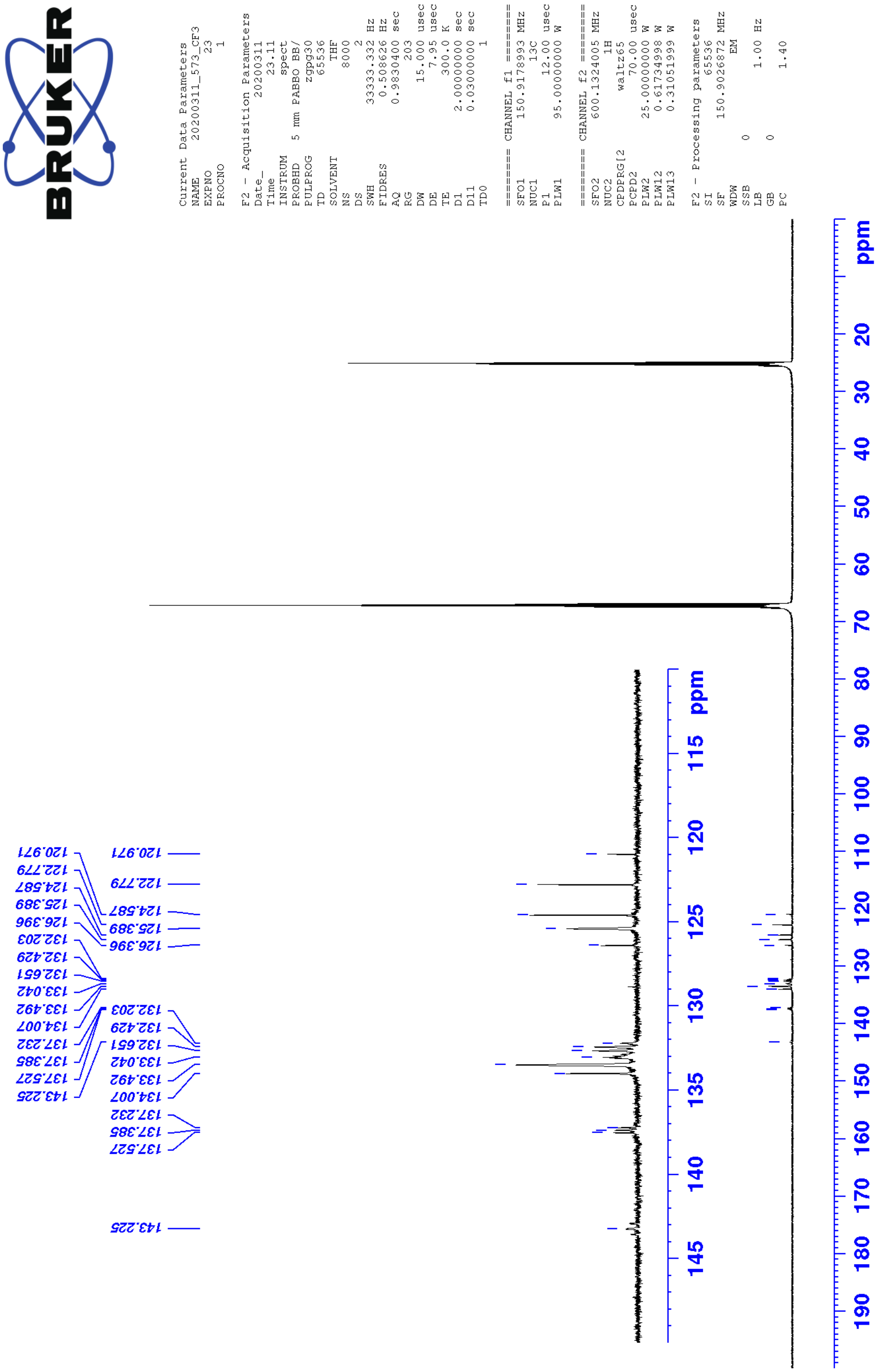

Supplementary Fig. 13 $^{31}P\{^{1}H\}$ NMR spectra of $[Rh(\mu\text{-}Cl)(CF_3\text{-dppbz})]_2$ (8) (THF-d_8).

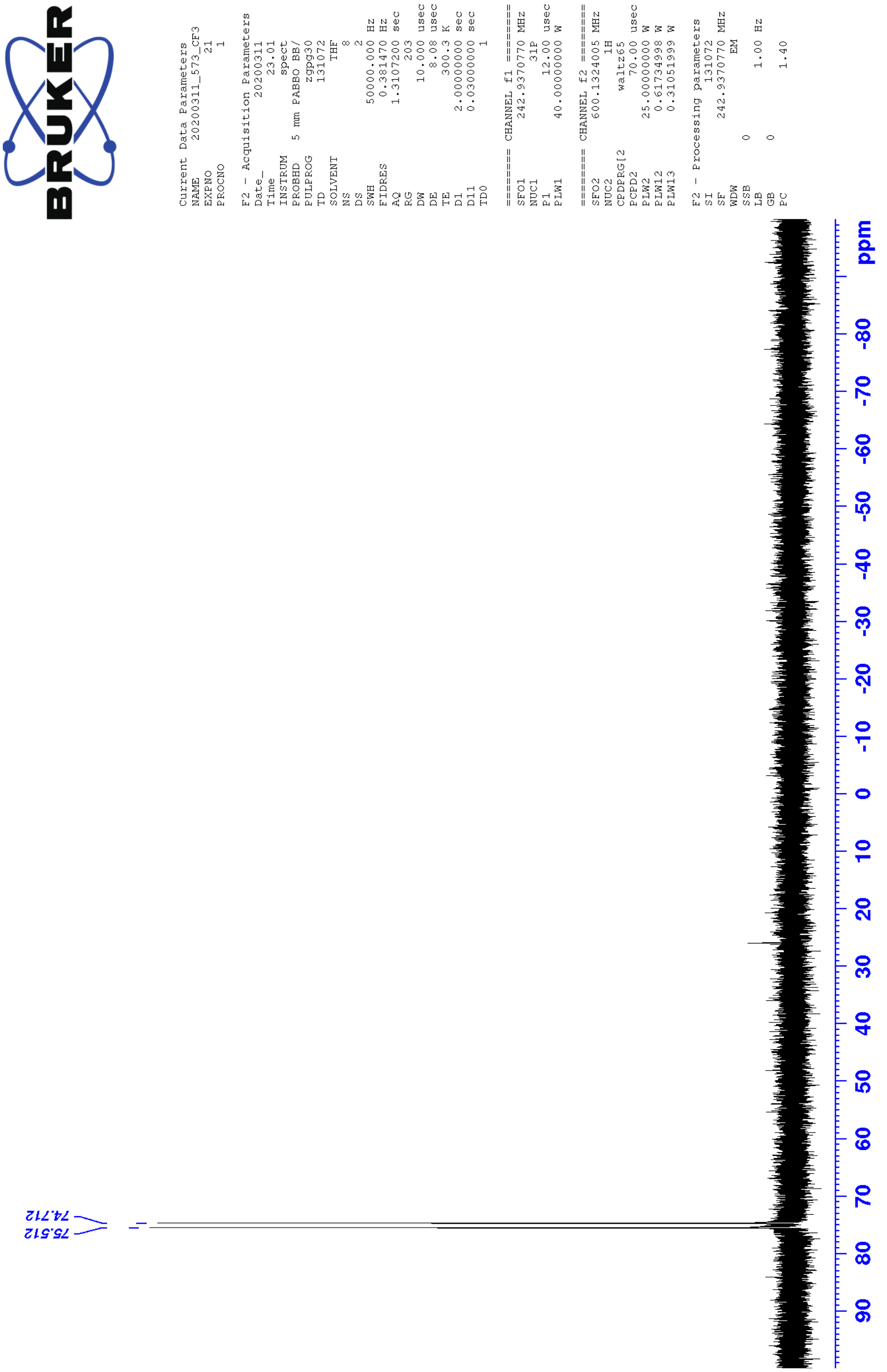

Supplementary Fig. 14 ^{1}H NMR spectra of [Rh(μ-Cl)(MeO-dppbz)]$_2$ (9) (THF-d_8).

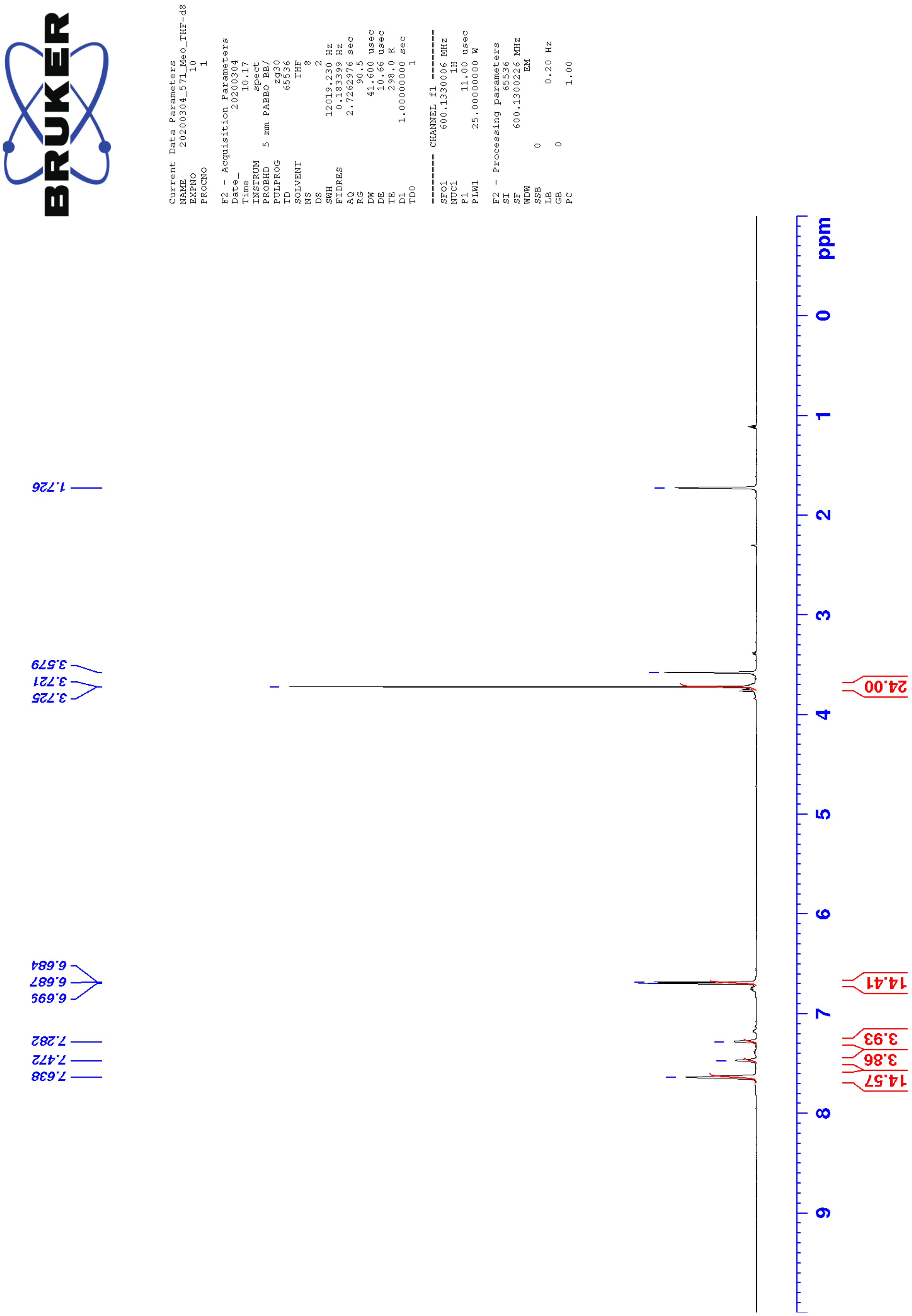

Supplementary Fig. 15 $^{13}C\{^{1}H\}$ NMR spectra of [Rh(μ-Cl)(MeO-dppbz)]$_2$ (9) (THF-d_8).

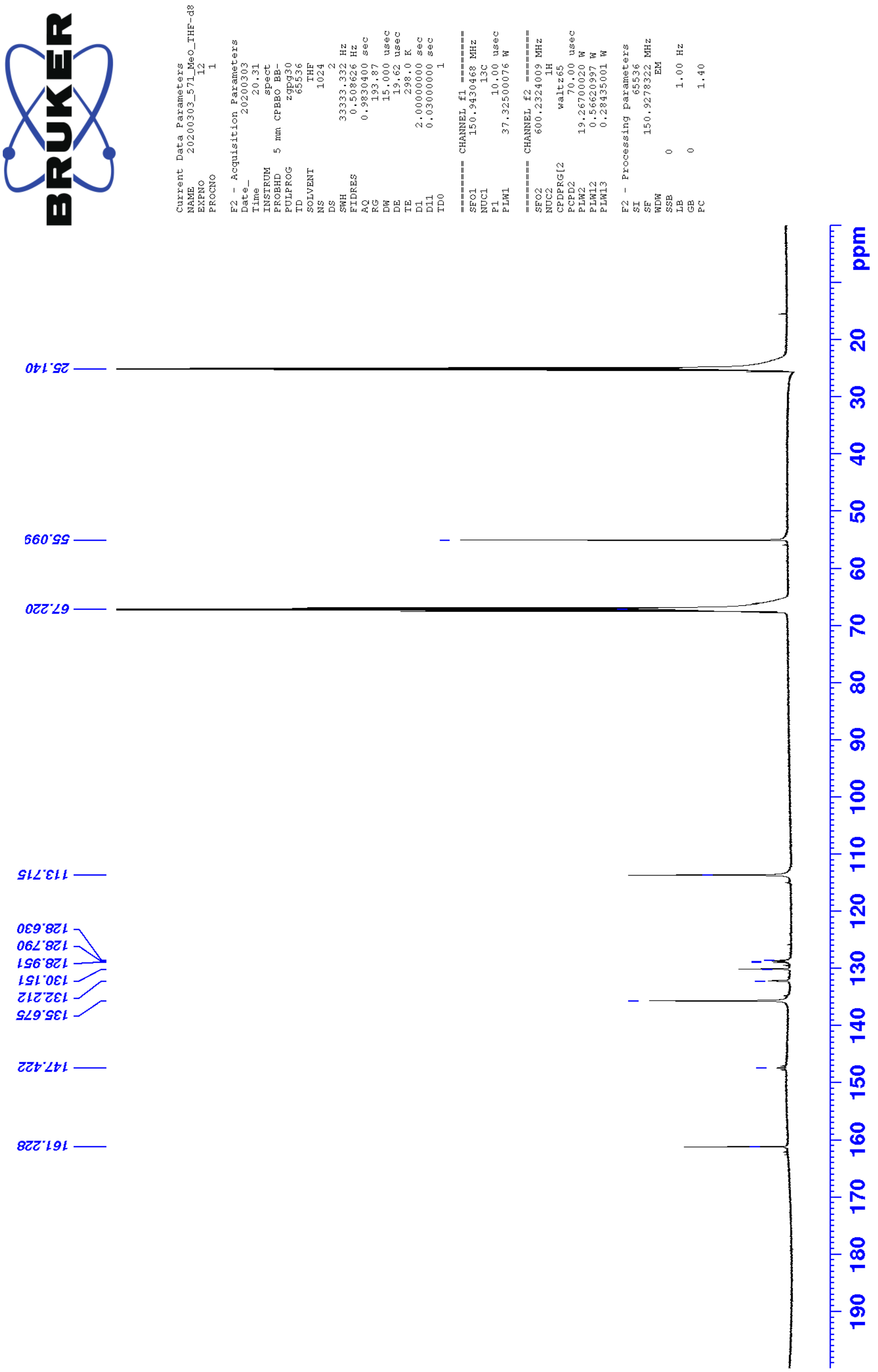

Supplementary Fig. 16 $^{31}P\{^{1}H\}$ NMR spectra of $[Rh(\mu\text{-}Cl)(MeO\text{-}dppbz)]_2$ (9) (THF-d_8).

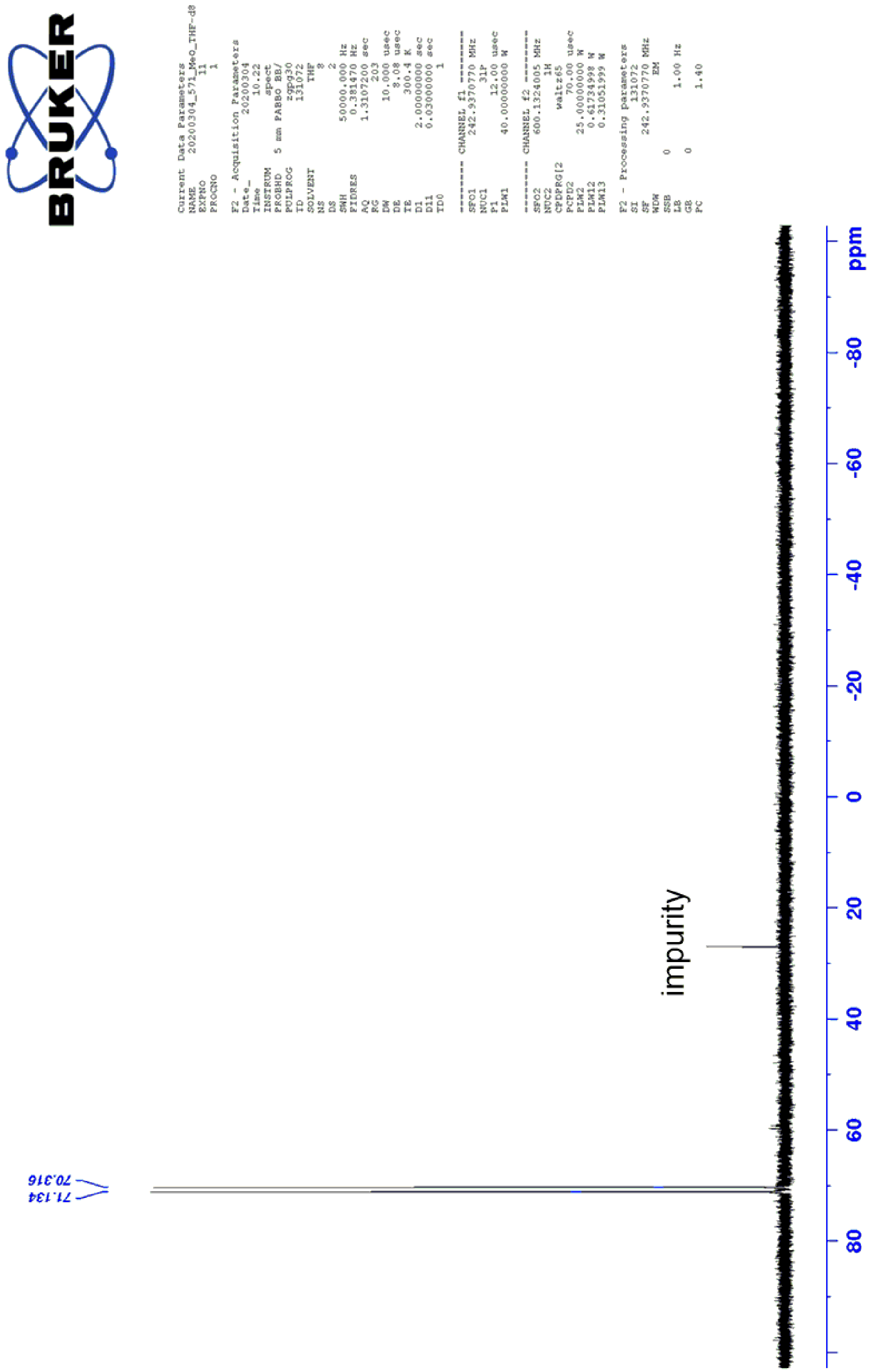

Supplementary Fig. 17 ^{1}H NMR spectra of $[Rh(\mu\text{-}Cl)(dppbz^{OMe})]_2$ (10) (THF-d_8).

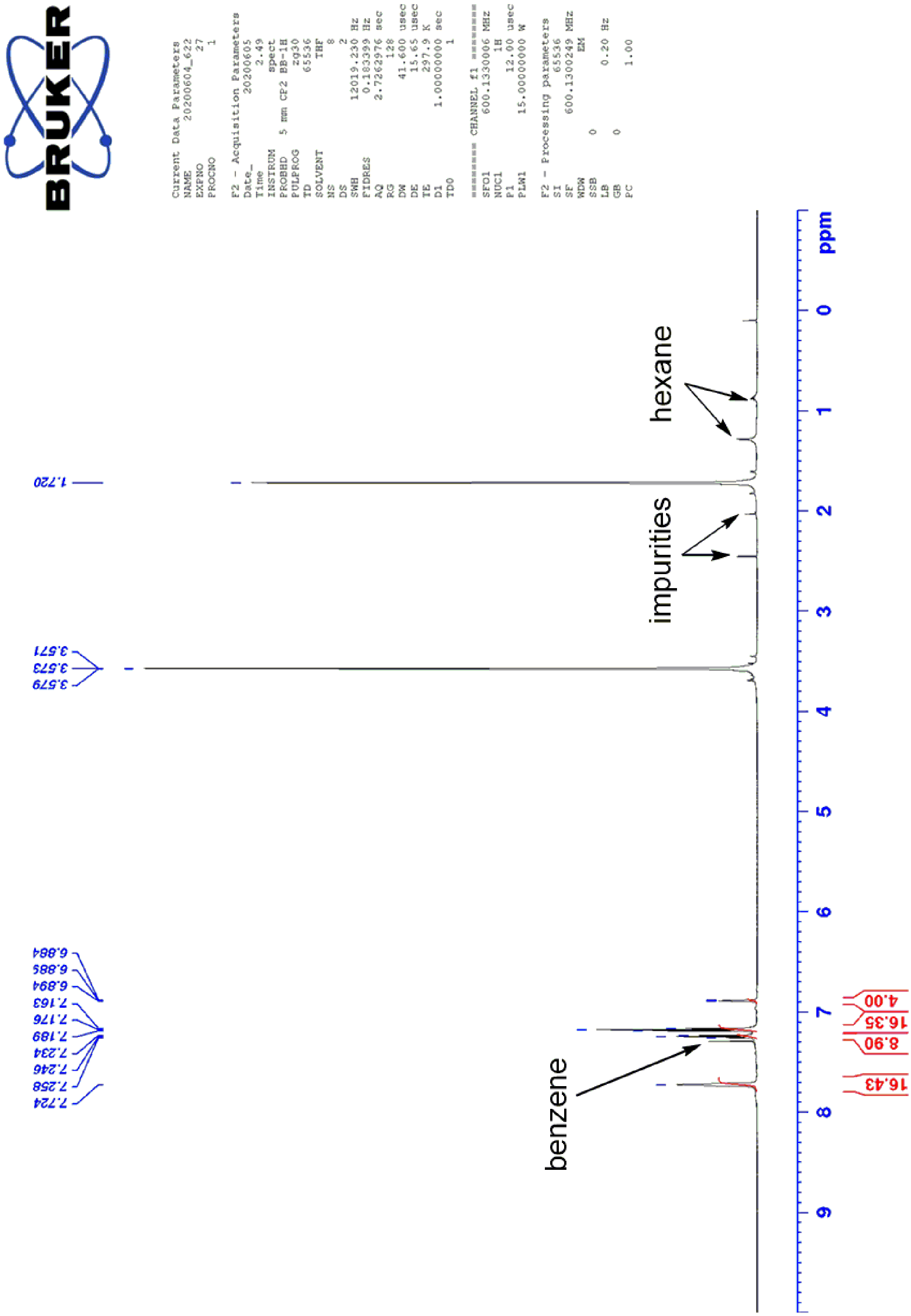

Supplementary Fig. 18 $^{13}C\{^{1}H\}$ NMR spectra of [Rh(μ-Cl)(dppbz^{OMe})]$_2$ (10) (THF-d_8).

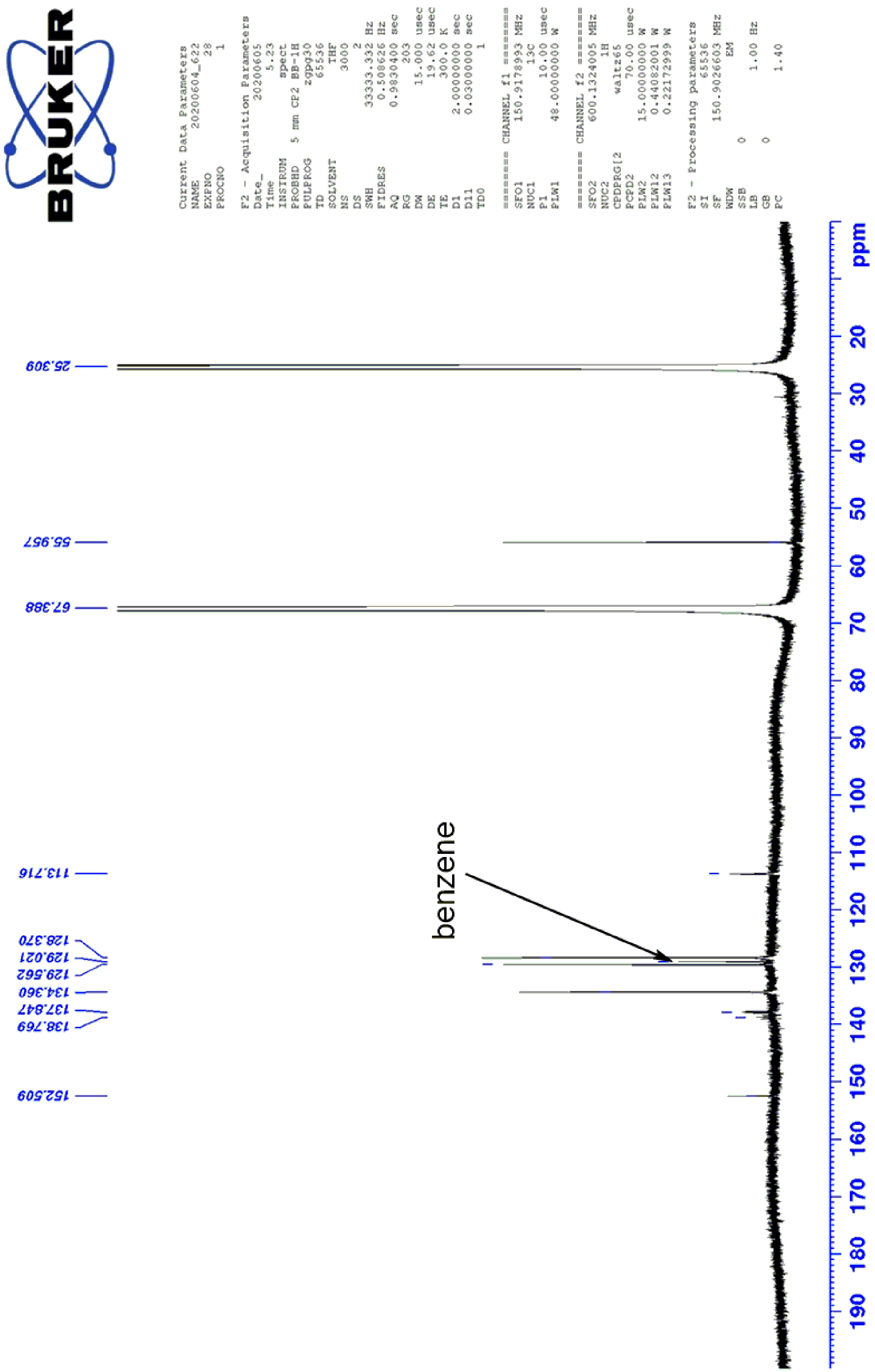

Supplementary Fig. 19 $^{31}P\{^{1}H\}$ NMR spectra of [Rh(μ-Cl)(dppbz^{OMe})]$_2$ (10) (THF-d_8).

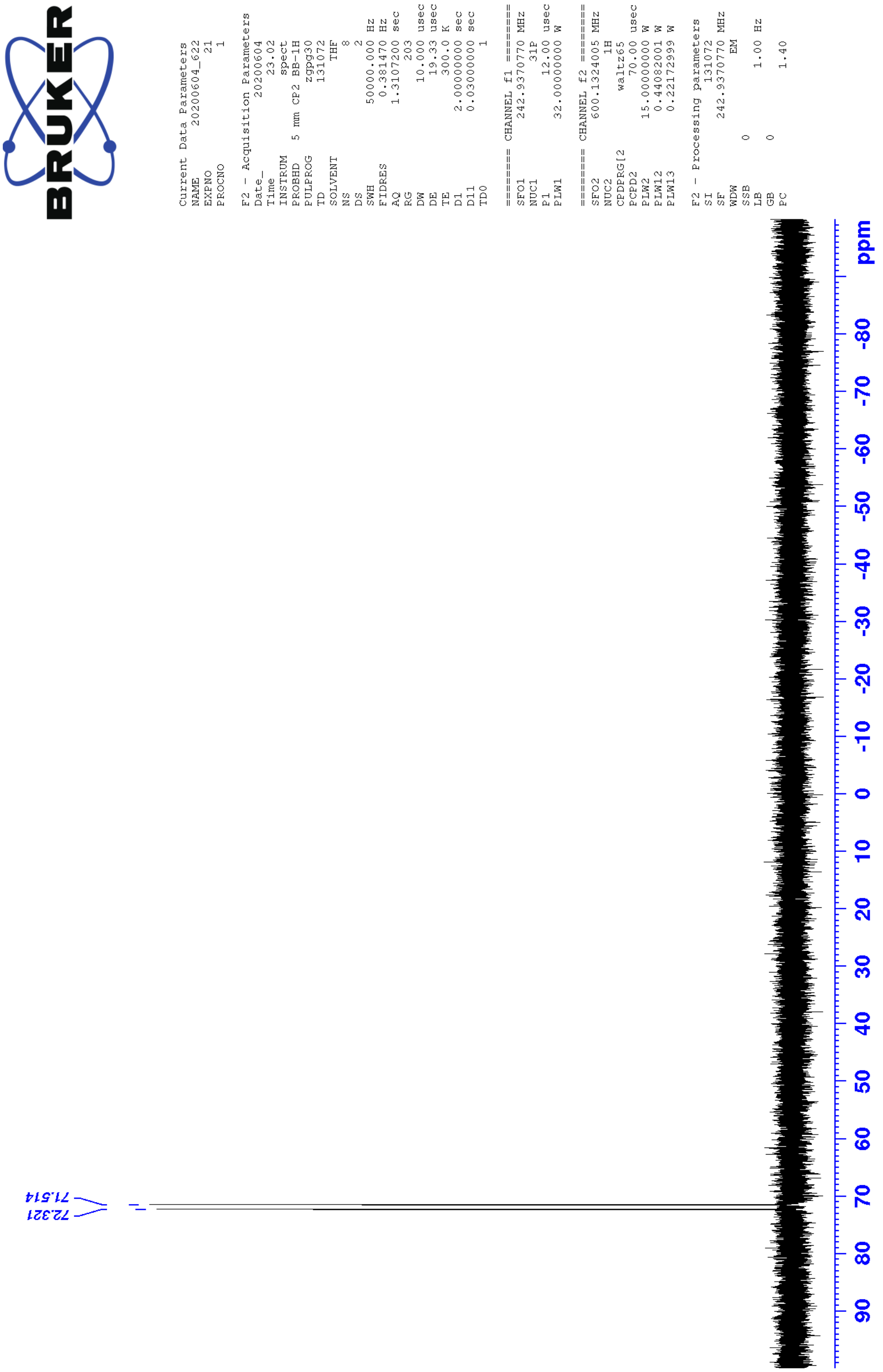

Supplementary Fig. 20 ^{1}H NMR spectra of [Rh(μ-Cl)(dppbz^{F})]$_2$ (11) (THF-d_8).

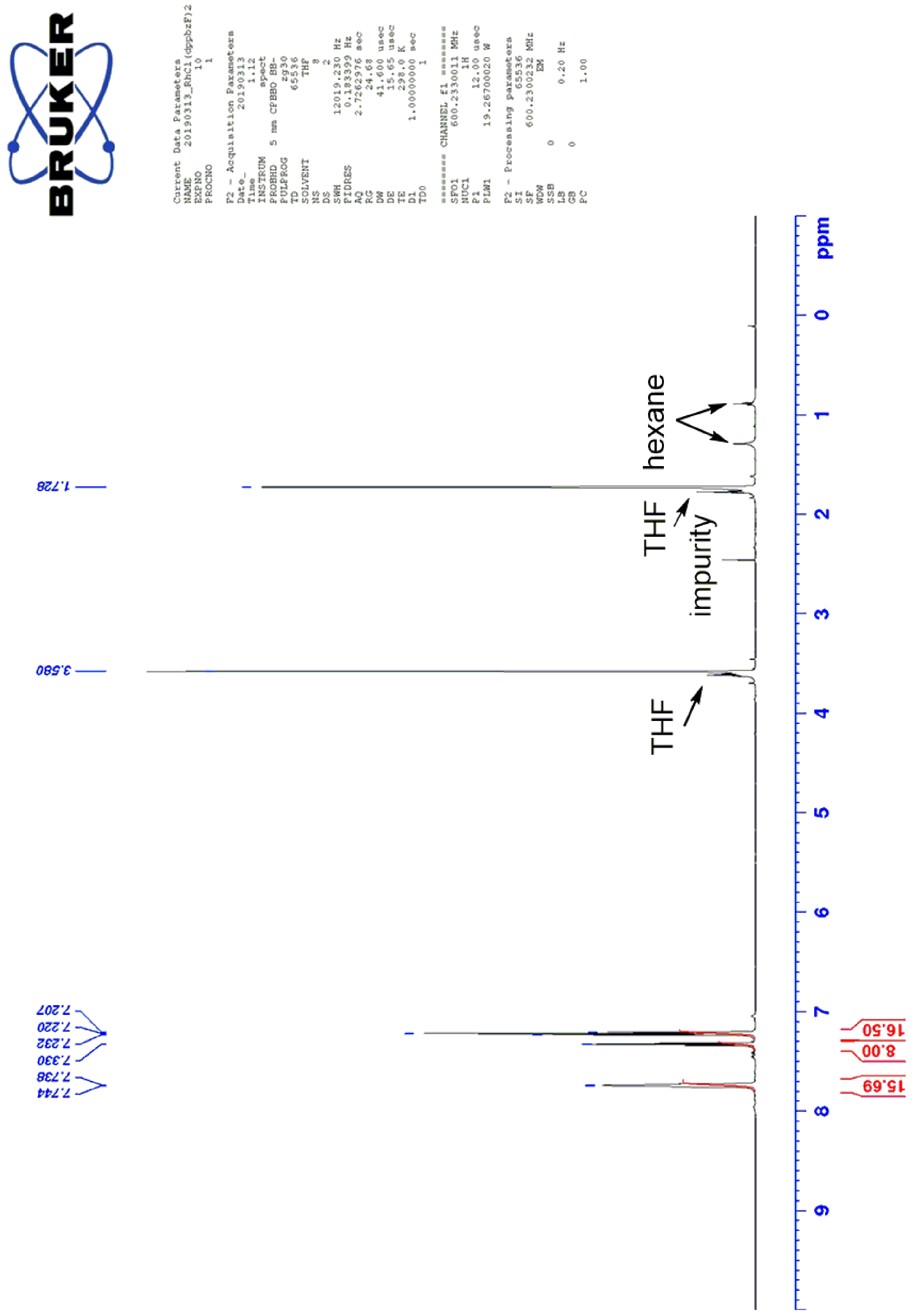

Supplementary Fig. 21 $^{13}C\{^{1}H\}$ NMR spectra of [Rh(μ-Cl)(dppbz^{F})]$_2$ (11) (THF-d_8).

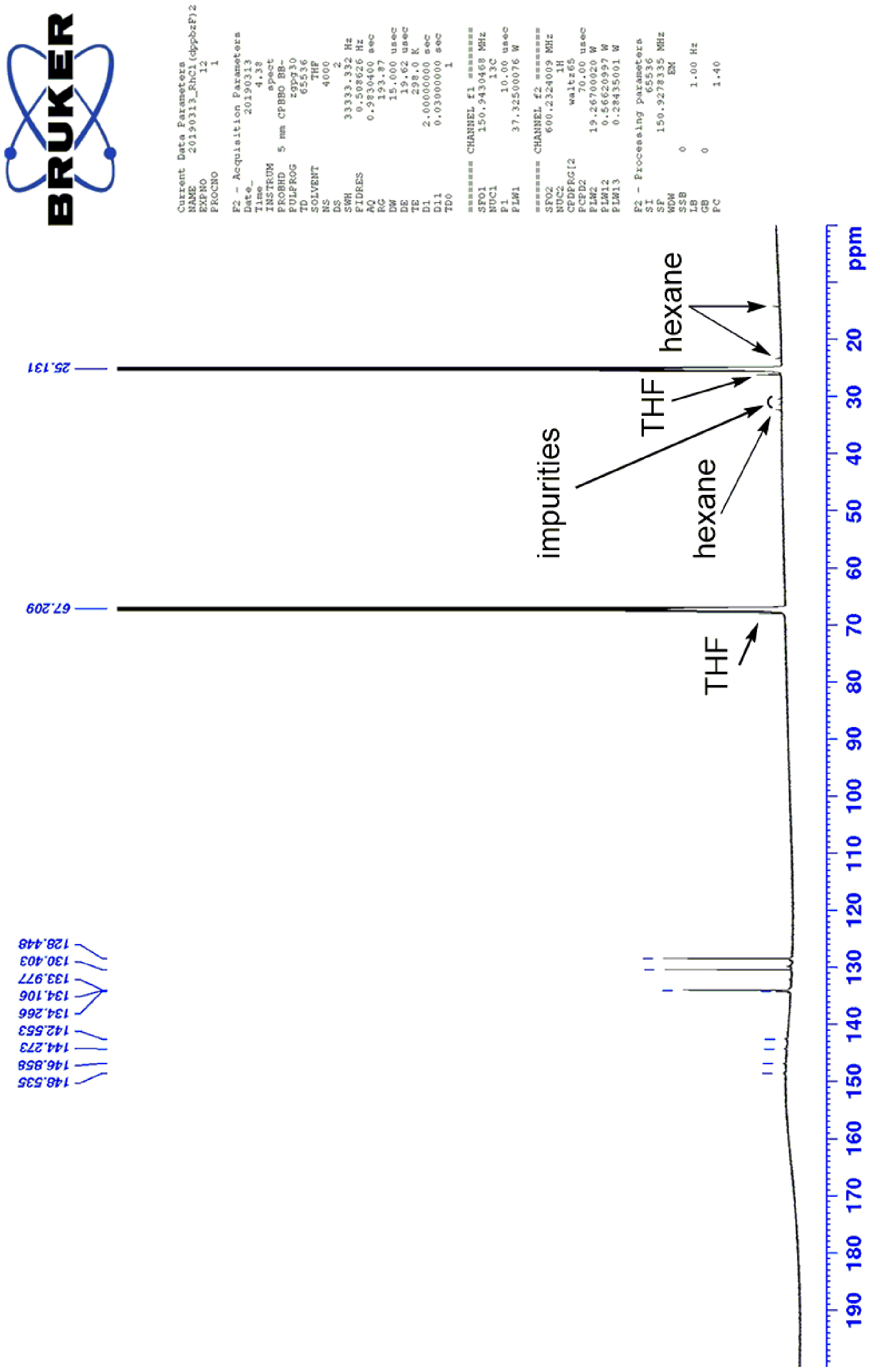

Supplementary Fig. 22 $^{31}P\{^{1}H\}$ NMR spectra of $[Rh(\mu\text{-}Cl)(dppbz^{F})]_2$ (11) (THF-d_8).

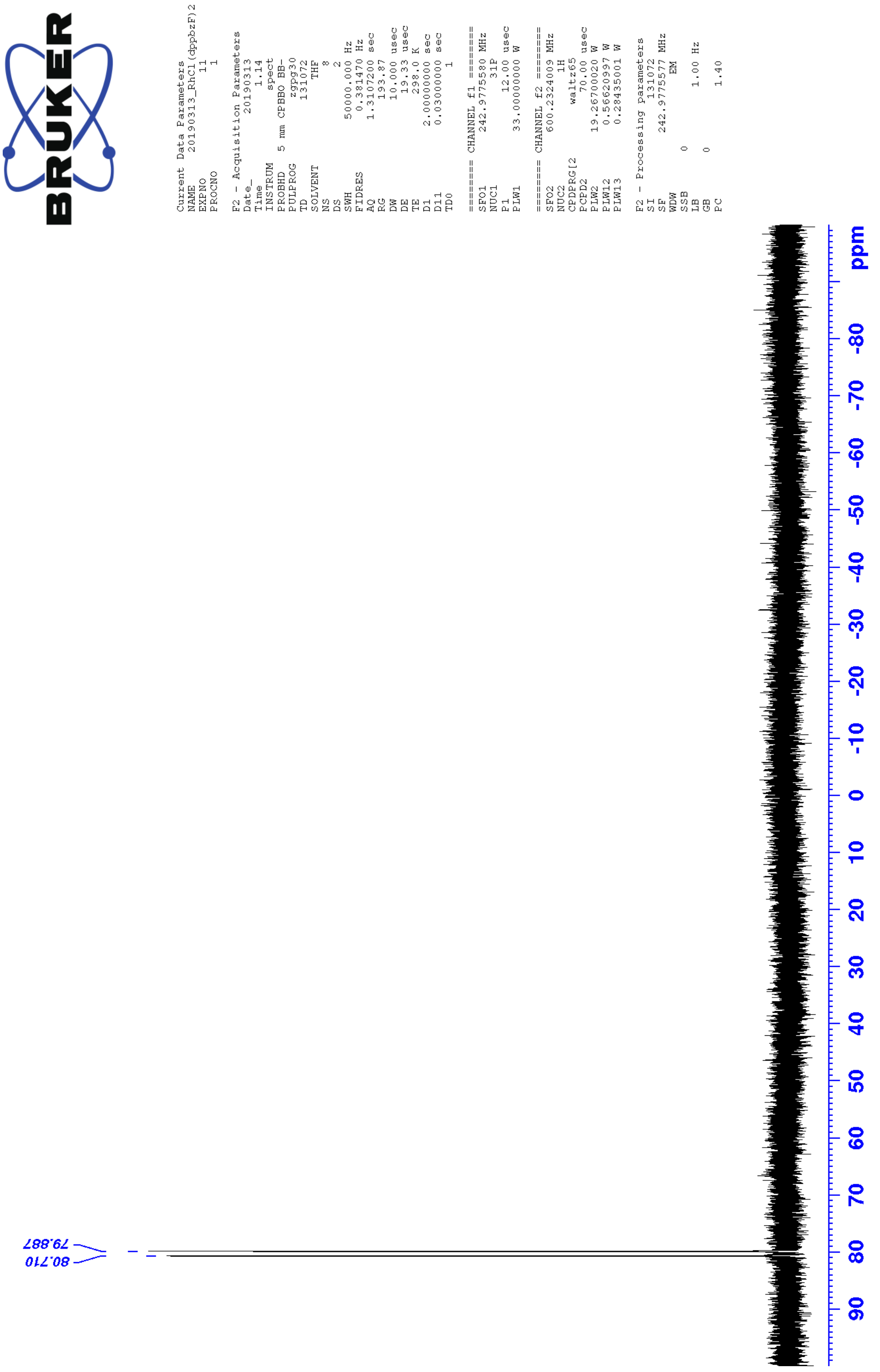

Supplementary Fig. 23 ^{1}H NMR spectra of [Rh(π-allyl)Cl_2(dpppbz^{F})] (12) (CD_2Cl_2).

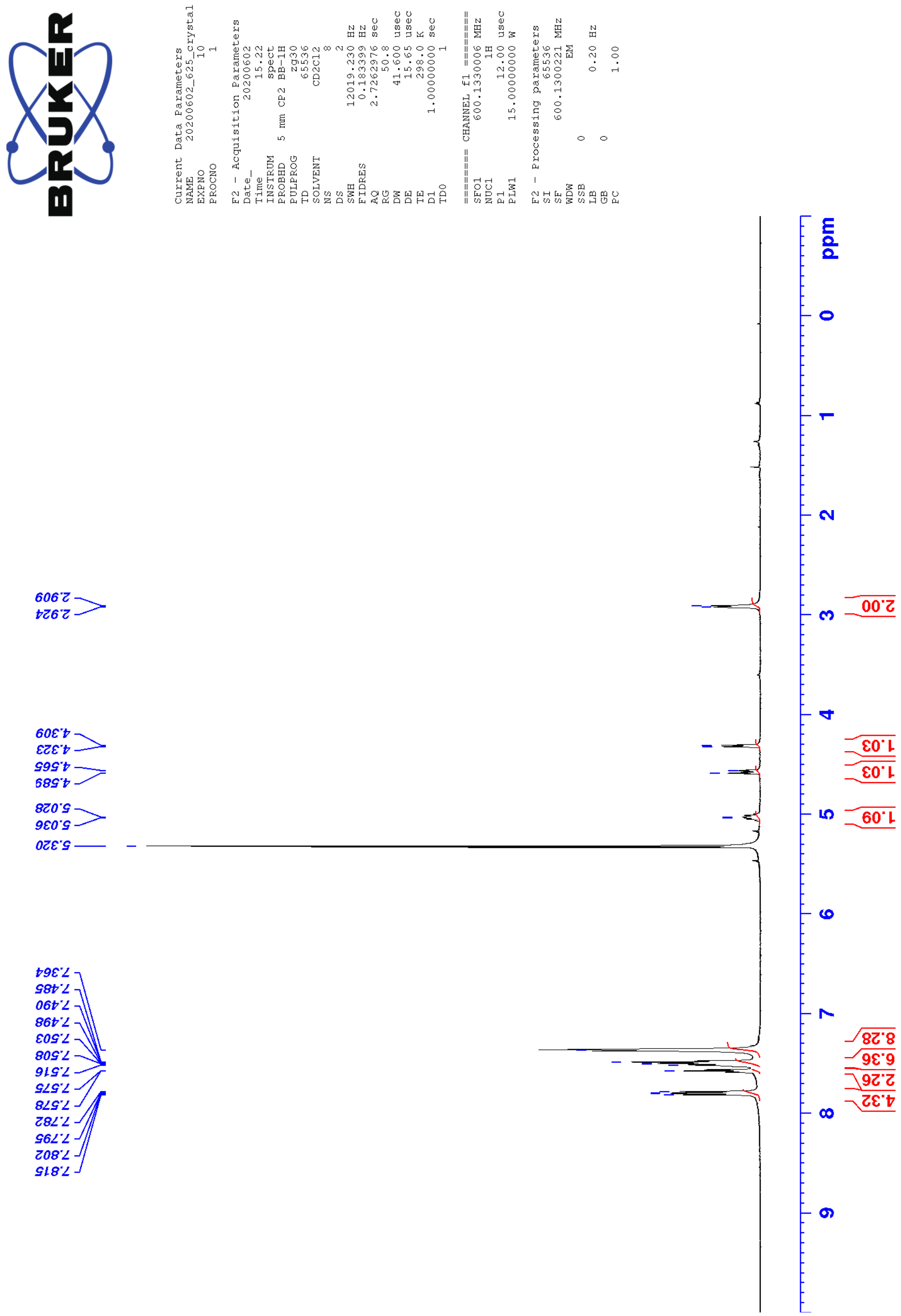

Supplementary Fig. 24 $^{13}C\{^1H\}$ NMR spectra of [Rh(π-allyl)Cl_2(dpppbz^F)] (12) (CD_2Cl_2).

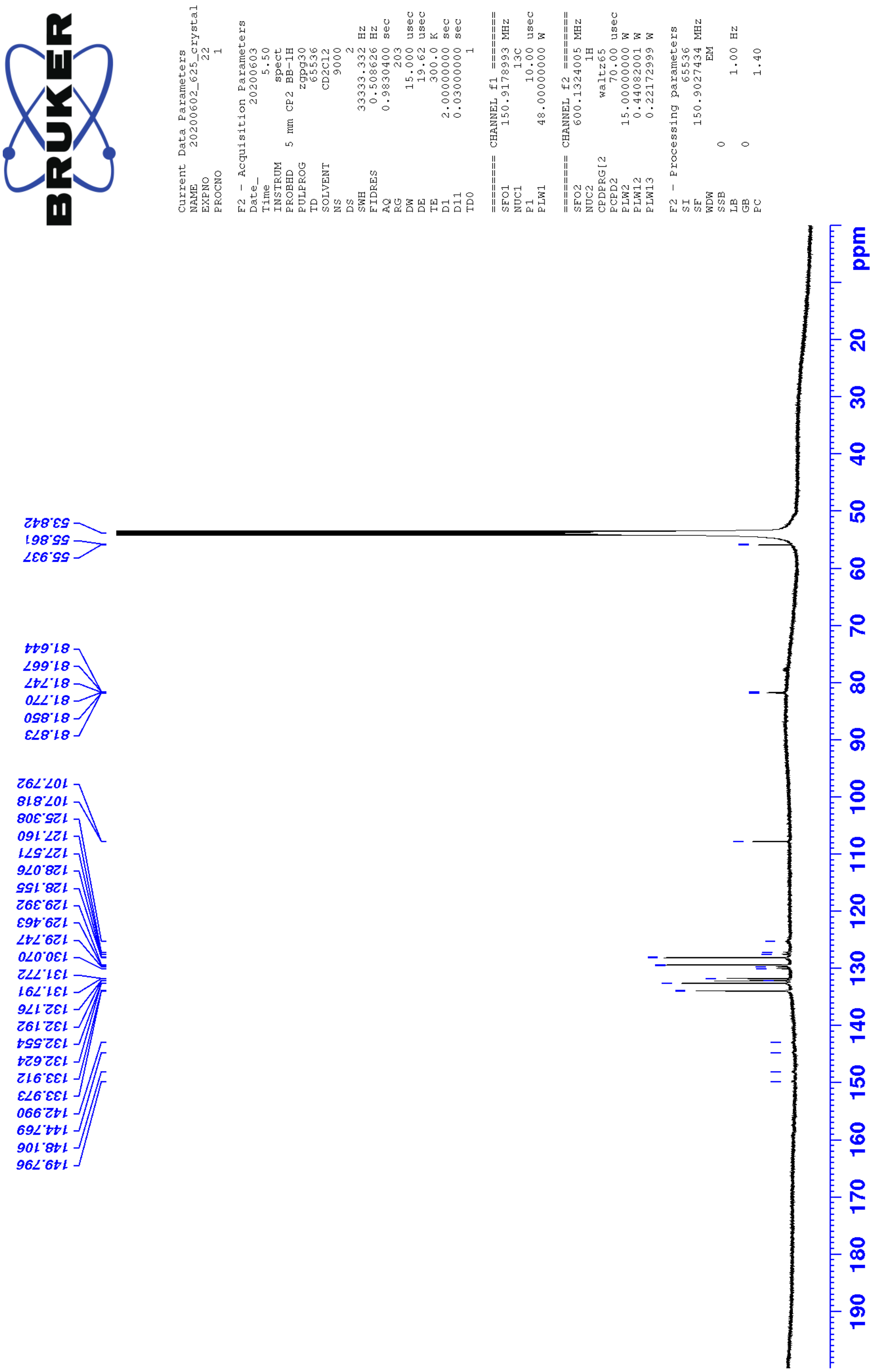

Supplementary Fig. 25 $^{31}P\{^{1}H\}$ NMR spectra of [Rh(π-allyl)Cl_2(dpppbz^F)] (12) (CD_2Cl_2).

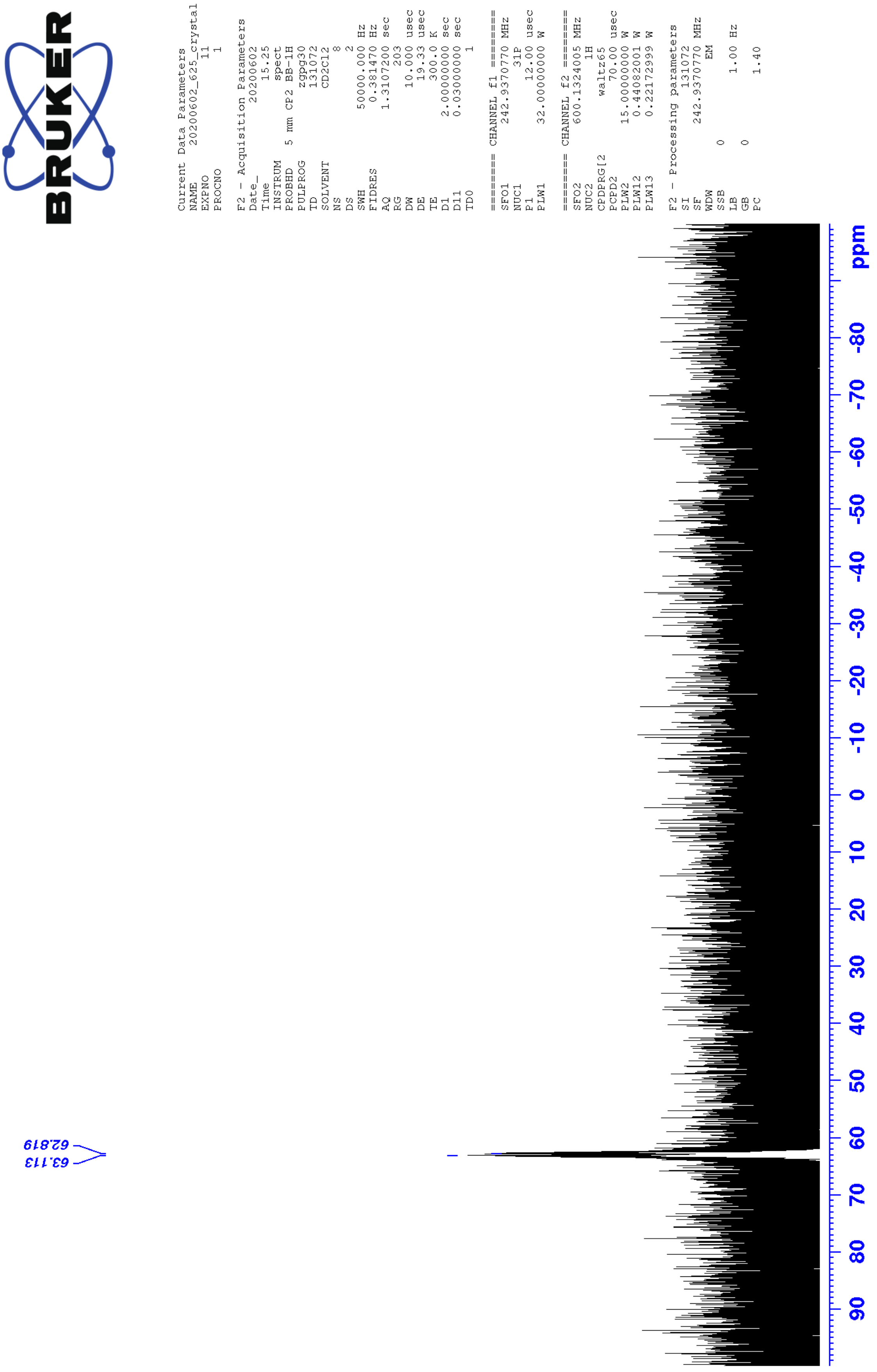

Supplementary Fig. 26 ^{1}H NMR spectra of [Rh(π-allyl)Cl_2(dppp)] (13) (CD_2Cl_2).

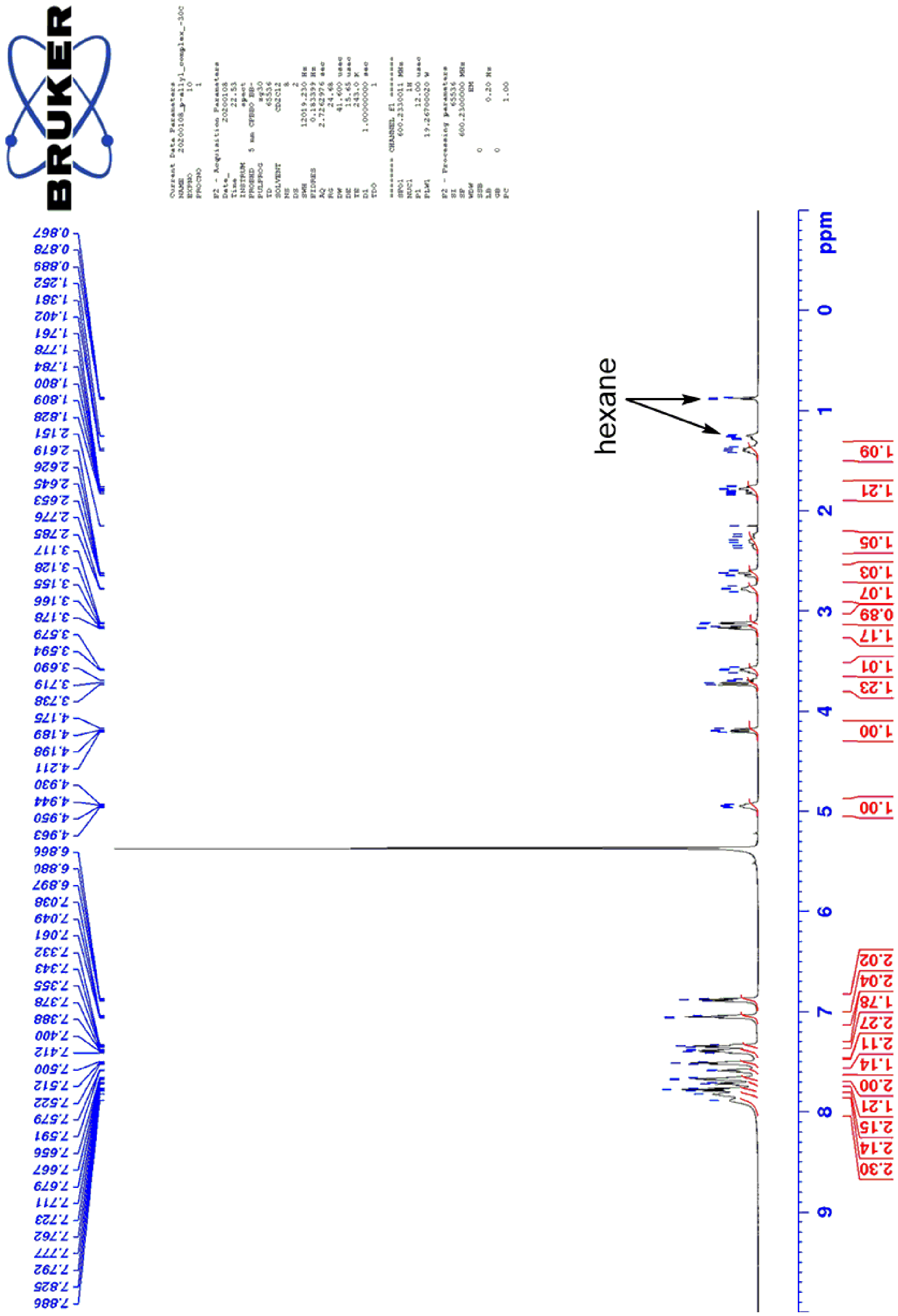

Supplementary Fig. 27 $^{13}C\{^{1}H\}$ NMR spectra of [Rh(π-allyl)Cl_2(dppp)] (13) (CD_2Cl_2).

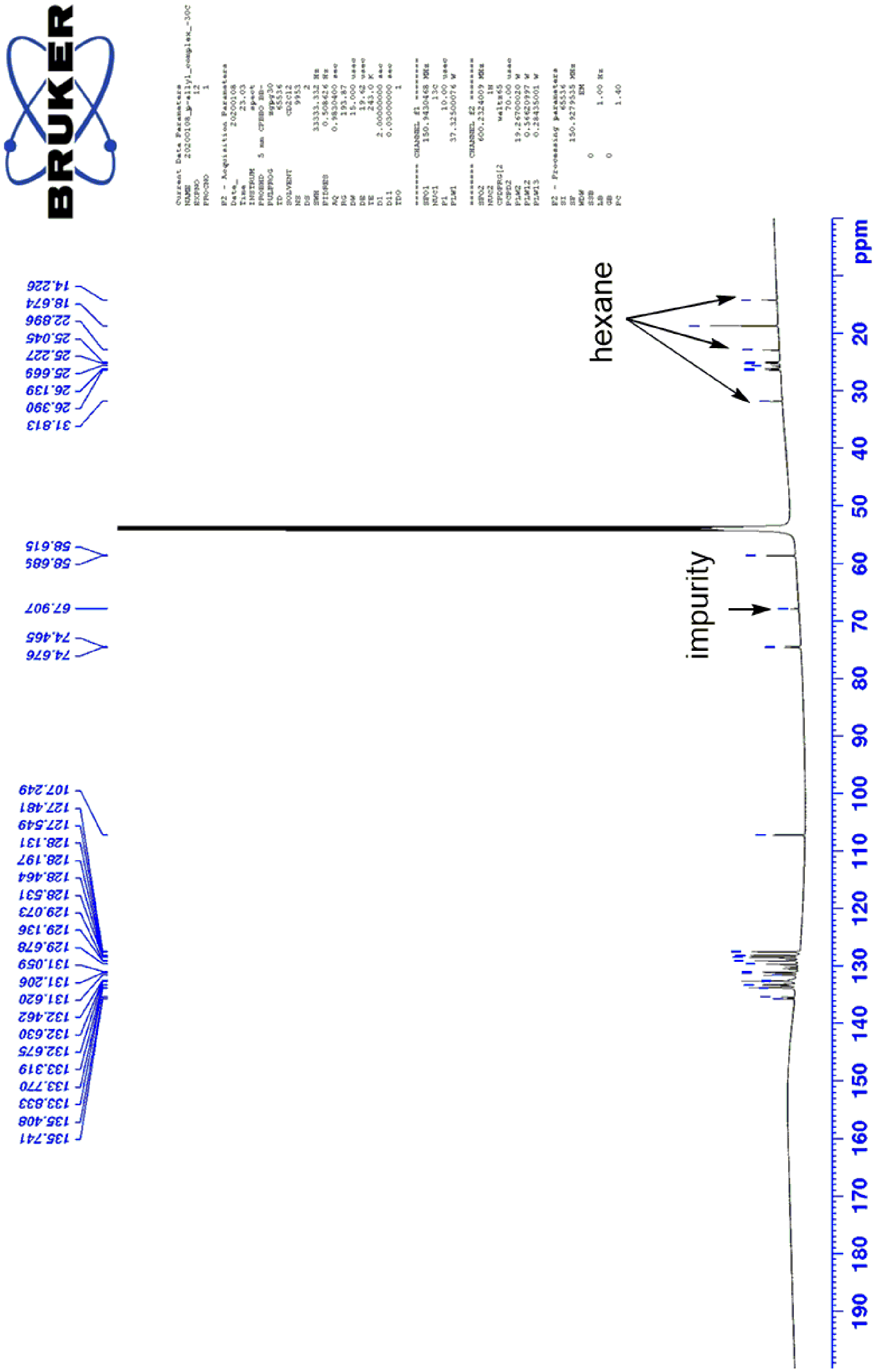

Supplementary Fig. 28 $^{31}P\{^{1}H\}$ NMR spectra of [Rh(π-allyl)Cl_2(dppp)] (13) (CD_2Cl_2).

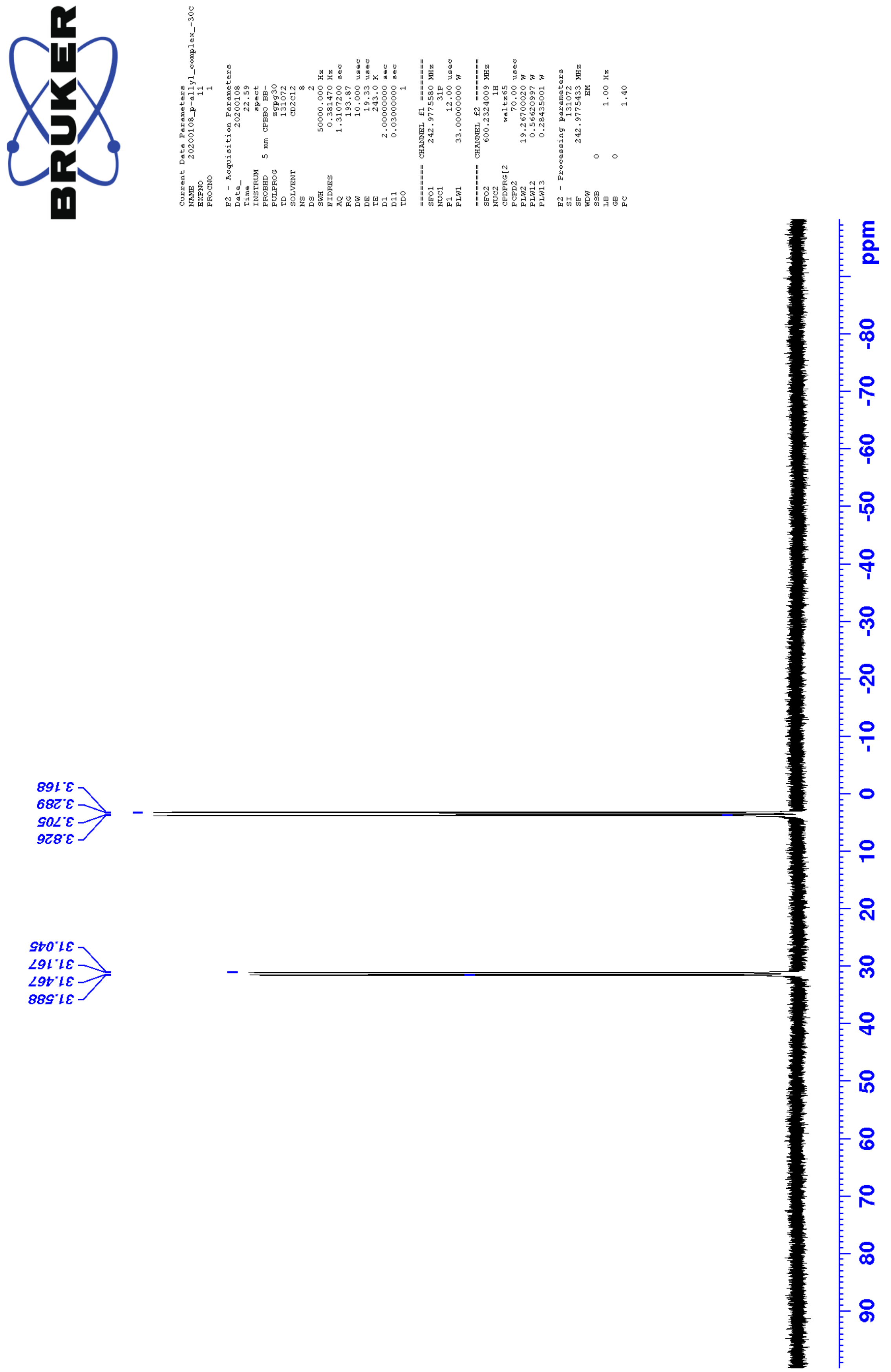

Supplementary Fig. 29 ^{1}H NMR spectra of [(dppp)Rh(Cl)(H)($SiCl_3$)] (14) (C_6D_6) .

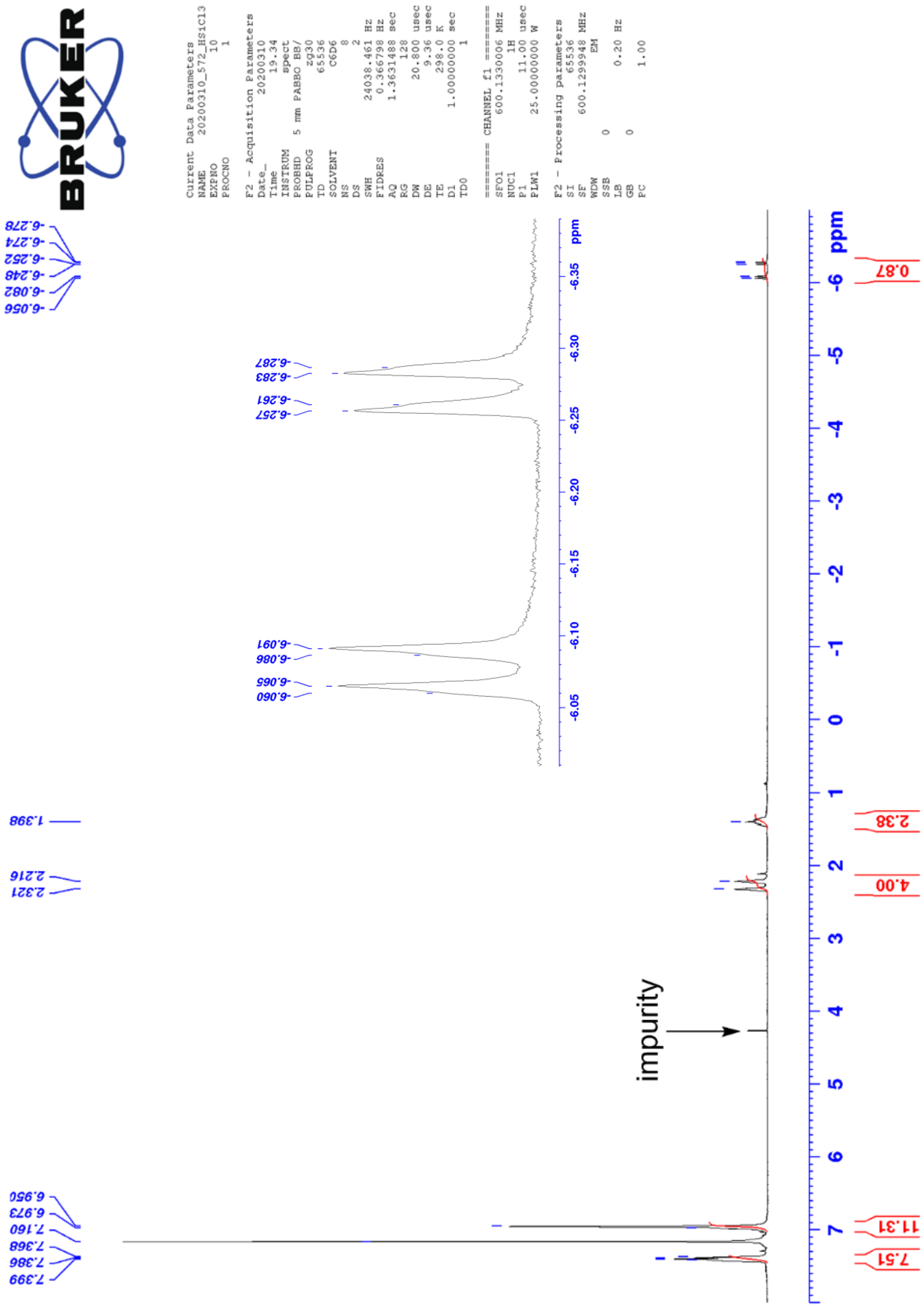

Supplementary Fig. 30 $^{13}C\{^{1}H\}$ NMR spectra of [(dppp)Rh(Cl)(H)($SiCl_3$)] (14) (C_6D_6).

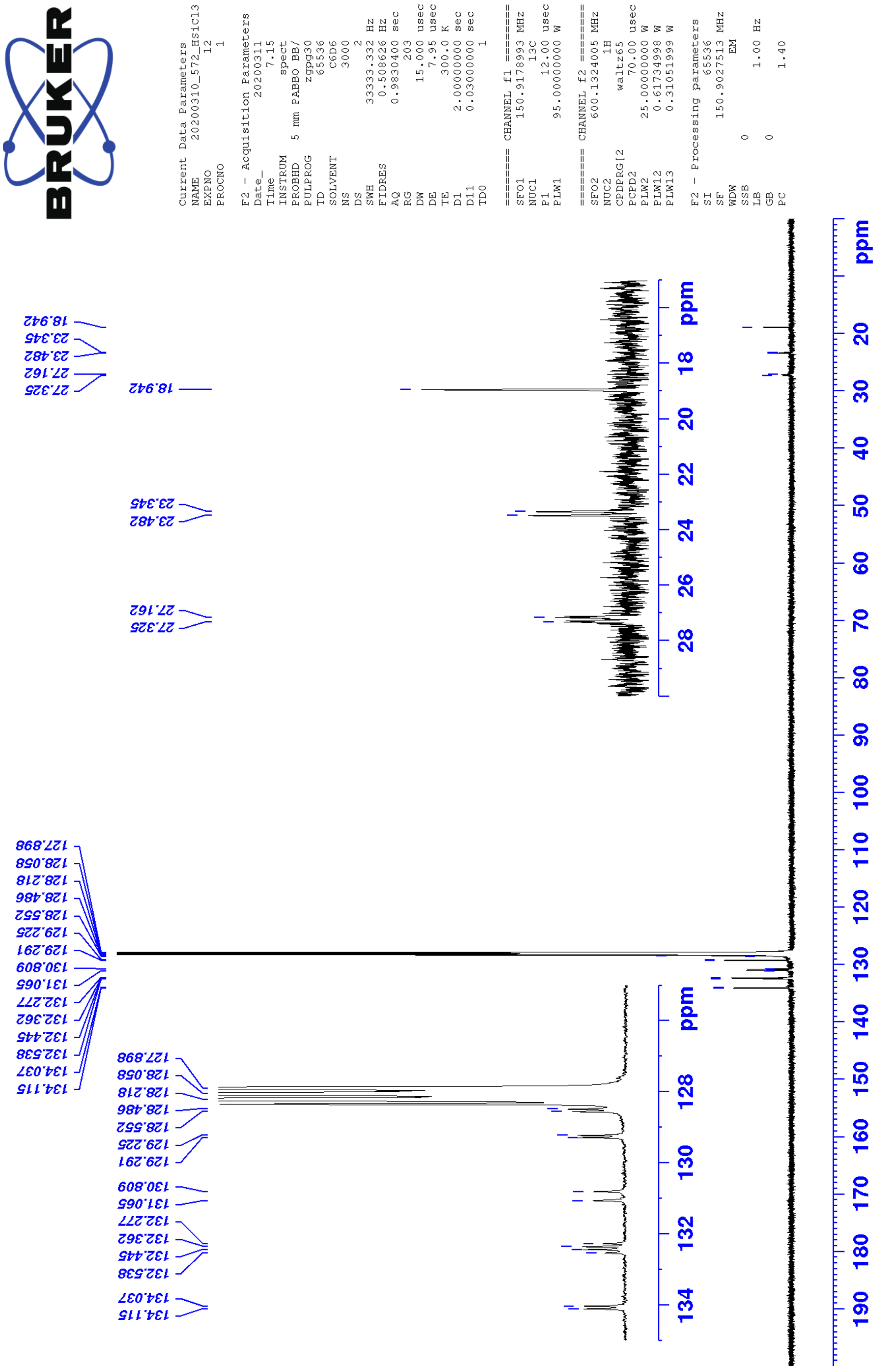

Supplementary Fig. 31 $^{31}P\{^1H\}$ NMR spectra of [(dppp)Rh(Cl)(H)(SiCl$_3$)] (14) (C_6D_6).

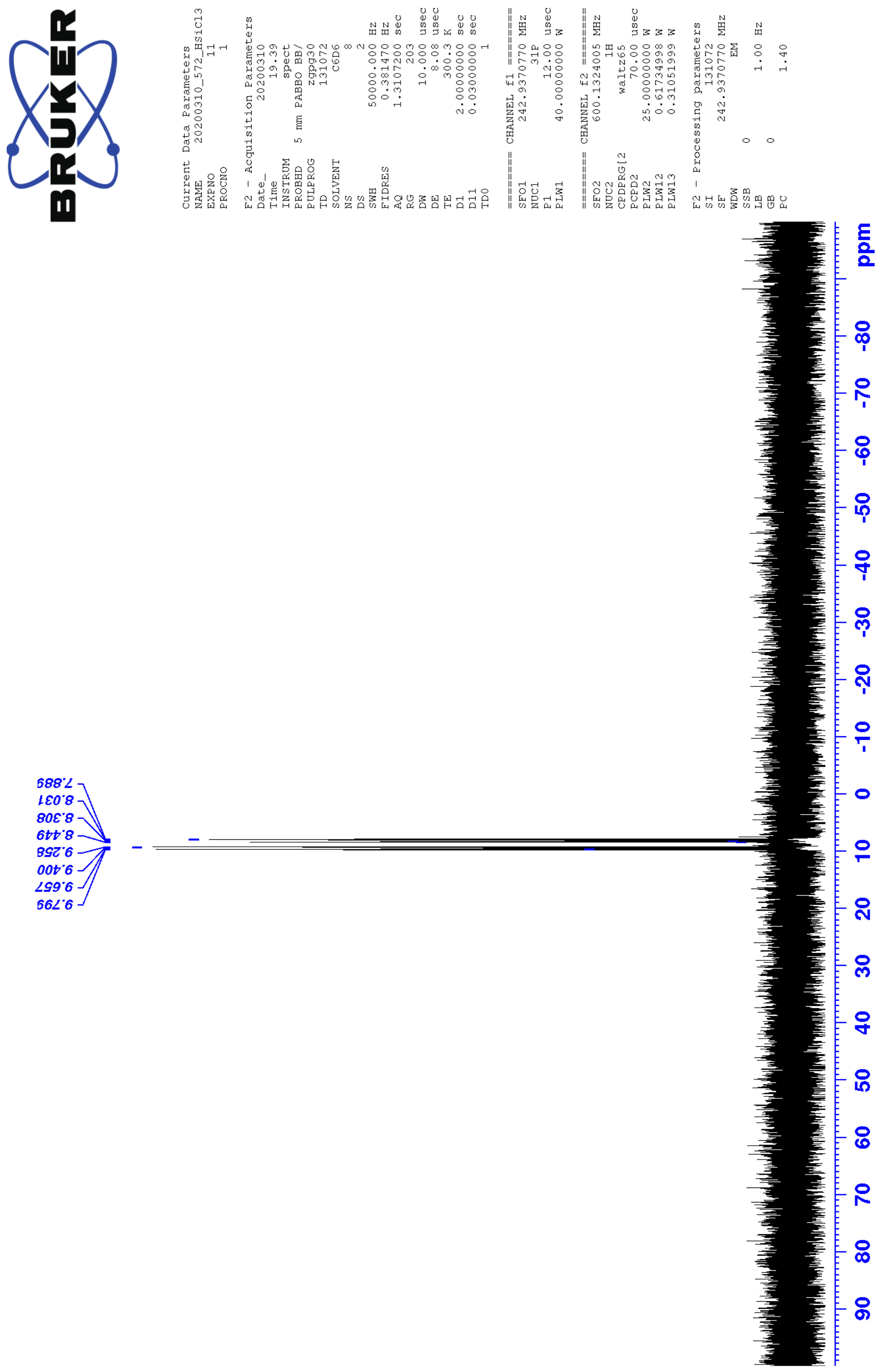

Supplementary Fig. 32 ^{1}H NMR spectrum of trichloro(3-chloropropyl)silane (1) (Fig 5, C_6D_6).

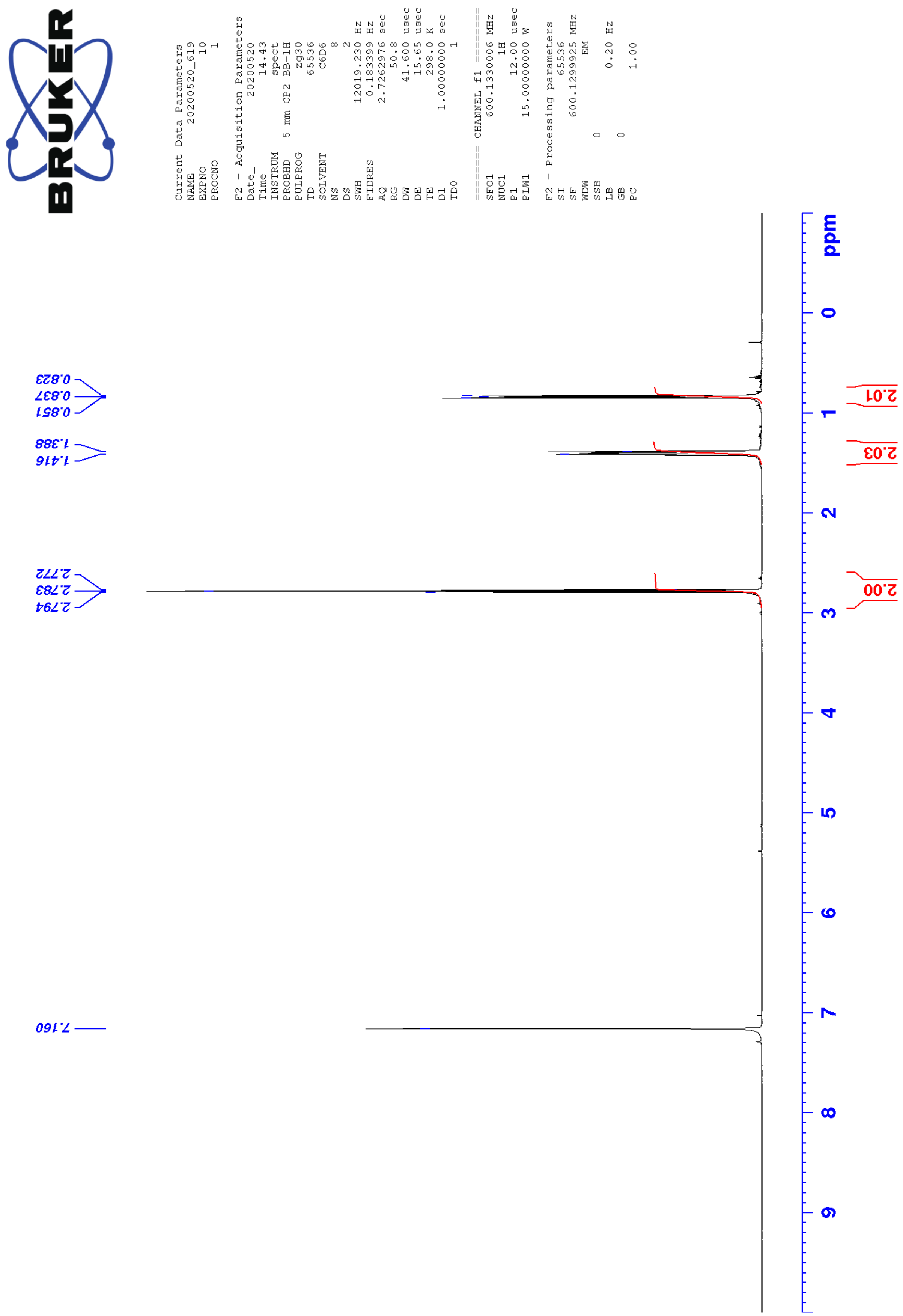

Supplementary references

1 Eller P.G., & Meek D. W. Syntheses of new tetrafluoroaryl derivatives of phosphorus and sulfur. *J. Organomet. Chem.* **22**, 631–636 (1970).

2 Ito S, Itoh T. & Nakamura M. Diastereoselective Carbometalation of oxa - and azabicyclic alkenes under iron catalysis. *Angew. Chem. Int. Ed.* **50**, 454–457 (2011).

3 Takata T., Nishikawa D., Hirano K. & Miura M. Synthesis of α - aminophosphines by copper - catalyzed regioselective hydroamination of cinylphosphines. *Chem. Eur. J.*, **24**, 10975–10978 (2018).

4 Choung R. et al. Ditopic ligands featuring [P,S], [P,P] or [P,B] chelating pockets housed on a protected *o*-hydroquinone core *J. Organomet. Chem.* 724, 45–50 (2013).

5 Park S., Kim B. G., Göttker-Schnetmann I. & Brookhart M. Redistribution of trialkyl silanes catalyzed by iridium silyl complexes. *ACS Catal.* **2**, 307-316 (2012).

6 Inokuchi H. et al. *The 4th Series of Experimental Chemistry*. **8**, 337–338 (Maruzen, Tokyo, 1990).

7 Heller D. et al. New pentacoordinated rhodium species as unexpected products during the In situ-generation of dimeric diphosphine-rhodium neutral catalysts. *Chem. Eur. J.* **20**, 14721–14728 (2014).

8 Heller D. Oxidative addition of CH_2Cl_2 to neutral dimeric rhodium diphosphine complexes. *J. Organomet. Chem.*, **871**, 178–184 (2018).

9 Riener K., Meister T. K., Gigler P., Herrmann W. A. & Kühn F. E. Mechanistic insights into the iridium-catalyzed hydrosilylation of allyl compounds. *J. Catal.* **331** 203–209 (2015).

10 Srinivas V., Nakajima Y., Sato K. & Shimada S. Iridium-catalyzed hydrosilylation of sulfur-containing olefins. *Org. Lett.* **20**, 12–15 (2018).

11 Igarashi M. et al. Ir-catalyzed hydrosilylation reaction of allyl acetate with octakis(dimethylsiloxy)octasilsesquioxane and related hydrosilanes. *J. Organomet. Chem.* **752**, 141–146 (2014).

12 Matsumoto K., Nakajima Y., Sato K., & Shimada S. WO 2017154846A (2017).

13 Xie X. et al. Iridium-catalyzed hydrosilylation of unactivated alkenes: scope and application to late-stage functionalization. *J. Org. Chem.* **84**, 1085–1093 (2019).

14 Mannu A., Vlahopoulou G., Kubis C. & Drexler H.-J. Synthesis and characterization of [Rh(PP)(PP)]X complexes (PP = DPPE or DPPP, X = Cl^{-} or BF_4^{-}). Phosphine exchange and reactivity in transfer hydrogenation conditions. *J. Organomet. Chem.* **885**, 59–64 (2019).

15 Bianchini C., Elsevier C. J., Emsting J. M., Peruzzini M. & Zanobini F. Control of the Bridgehead Donor Atom in the Tripodal Ligand over Oxidative Addition of $Au(PPhs)^{+}$ to $[X(CH_2CH_2PPh_2)_3RhH]$ (X = N, P). X-ray Diffraction and Multinuclear (^{103}Rh, ^{31}P, and ^{1}H) NMR Studies. *Inorg. Chem.* **34**, 84-92 (1995).
